# Enhanced Leaf Cooling Is a Pathway to Heat Tolerance in Common Bean

**DOI:** 10.3389/fpls.2020.00019

**Published:** 2020-02-28

**Authors:** Chetan R. Deva, Milan O. Urban, Andrew J. Challinor, Pete Falloon, Lenka Svitákova

**Affiliations:** ^1^ Climate Impacts Group, Institute for Climate and Atmospheric Science, School of Earth and Environment, University of Leeds, Leeds, United Kingdom; ^2^ The International Center for Tropical Agriculture (CIAT), Cali, Colombia; ^3^ The Met Office Hadley Centre, Exeter, United Kingdom; ^4^ Department of Experimental Plant Biology, Faculty of Science, Charles University, Prague, Czechia

**Keywords:** heat tolerance, common bean, leaf temperature depression, VPD, plant breeding, modeling, climate change adaptation

## Abstract

Common bean is the most consumed legume in the world and an important source of protein in Latin America, Eastern, and Southern Africa. It is grown in a variety of environments with mean air temperatures of between 14°C and 35°C and is more sensitive to high temperatures than other legumes. As global heating continues, breeding for heat tolerance in common bean is an urgent priority. Transpirational cooling has been shown to be an important mechanism for heat avoidance in many crops, and leaf cooling traits have been used to breed for both drought and heat tolerance. As yet, little is known about the magnitude of leaf cooling in common bean, nor whether this trait is functionally linked to heat tolerance. Accordingly, we explore the extent and genotypic variation of transpirational cooling in common bean. Our results show that leaf cooling is an important heat avoidance mechanism in common bean. On average, leaf temperatures are 5°C cooler than air temperatures, and can range from between 13°C cooler and 2°C warmer. We show that the magnitude of leaf cooling keeps leaf temperatures within a photosynthetically functional range. Heat tolerant genotypes cool more than heat sensitive genotypes and the magnitude of this difference increases at elevated temperatures. Furthermore, we find that differences in leaf cooling are largest at the top of the canopy where determinate bush beans are most sensitive to the impact of high temperatures during the flowering period. Our results suggest that heat tolerant genotypes cool more than heat sensitive genotypes as a result of higher stomatal conductance and enhanced transpirational cooling. We demonstrate that it is possible to accurately simulate the temperature of the leaf by genotype using only air temperature and relative humidity. Our work suggests that greater leaf cooling is a pathway to heat tolerance. Bean breeders can use the difference between air and leaf temperature to screen for genotypes with enhanced capacity for heat avoidance. Once evaluated for a particular target population of environments, breeders can use our model for modeling leaf temperatures by genotype to assess the value of selecting for cooler beans.

## Introduction

Common bean (Phaseolus vulgaris) is the most consumed legume in the world (Araújo et al., 2015), and an important source of protein in tropical Latin America, Eastern, and Southern Africa (Beebe et al., 2011). Common bean is grown in a variety of environments with mean air temperatures of between 14°C and 35°C (Araújo et al., 2015). There are two major gene pools, Andean and Mesoamerican. Beans from the Andean gene pool are adapted to mid-higher altitudes (1,400–2,800 masl) and cooler temperatures, while beans from the Mesoamerican gene pool are adapted to low-mid altitudes (400–2,000 masl) (Araújo et al., 2015). Common bean is more sensitive to high temperatures than other legumes (Beebe et al., 2011), making breeding for heat tolerance an urgent priority as the climate continues to warm (Beebe et al., 2011).

Plants are described as being heat tolerant if they are able to maintain the capacity to grow and produce economic yields at high temperatures (Wahid et al., 2007). Some heat tolerant crops maintain photosynthesis under elevated temperatures by maintaining stomatal conductance (Porch and Hall, 2013). Keeping the stomata open at elevated temperatures sustains diffusion of CO2 into the leaves and enhances transpirational cooling (Porch and Hall, 2013). Plants that are able to maintain stomatal conductance at high temperatures are therefore better able to regulate their temperature (Porch and Hall, 2013; Prasad et al., 2017). It has been suggested that enhanced transpirational cooling may be a useful trait in identifying bean genotypes with the thermal plasticity to adapt to climate change (McClean et al., 2011). The magnitude of transpirational cooling has been used by plant breeders to screen for heat tolerance in spring wheat cultivars (Porch and Hall, 2013). We now turn to the evidence on the contribution of leaf/canopy cooling to heat avoidance in important food crops and the links between heat tolerance and leaf cooling.

Plants that have evolved in extreme environments are able to strongly regulate the temperature of their leaves, decoupling leaf and air temperatures. In cool alpine environments and humid tropical conditions, leaf temperature can exceed air temperature by as much as 20°C. In hot and dry desert conditions on the other hand, leaf temperature can be 20°C cooler than air temperature (Blonder and Michaletz, 2018). A recent review of the challenges facing field crops from rising temperatures identifies further research into the physiology of canopy cooling as a key priority (Prasad et al., 2017).

From an energy balance perspective, leaf thermoregulation is controlled by net radiation and evaporative cooling. The relationship between these variables is mediated by leaf thermal traits, including stomatal conductance, size, shape, absorptivity, and emissivity (Michaletz et al., 2016). Stomatal conductance responds to many internal and external factors that influence the rate of carbon assimilation and transpiration. In a simplified model of photosynthesis, when RubP carboxylase/oxygenase is unsaturated, stomata respond to the gradient between intercellular and ambient levels of carbon dioxide to maximize assimilation. Similarly, stomatal conductance is sensitive to the hydraulic gradient between the soil, stem, leaf, and atmosphere. When there is a lack of water, stomatal conductance decreases, reducing transpiration, and conserving water (Farquhar and Sharkey, 1982). Since high temperatures and drought often occur simultaneously, stomatal behavior points towards a potential trade-off between leaf cooling and water conservation in hot non-irrigated conditions.

Successful breeding of food crops able to avoid high temperatures through enhanced cooling requires an understanding of the magnitude and interspecies variation in transpirational cooling. There has been significant progress in understanding the role of transpirational cooling in temperature regulation of rice and wheat plants. Controlled experiments have shown that the difference between the temperature of the air and the reproductive organs of a rice plant is mediated by relative humidity (Weerakoon et al., 2008). This finding has been supported by experiments in field conditions. In hot and dry rice growing conditions in Senegal, the temperature of reproductive organs was found to be up to 9.5°C cooler than the air temperature, while in cooler and more humid conditions in the Philippines, reproductive organs were at times hotter than the air by 2°C (Julia and Dingkuhn, 2013). A large range in canopy temperature depression (CTD) has also been found in wheat plants. Under varying soil moisture conditions, the canopy temperature ranged from between 6°C cooler and 7°C warmer than the temperature of the air (Siebert et al., 2014). Though less well established, there is also a smaller body of evidence suggesting that transpirational cooling is an important mechanism for avoiding stress at high temperatures in potato, maize and a variety of legumes (Kumar et al., 2017a).

Transpirational cooling also varies within species. In rice and wheat, there is robust evidence of within species variation, which has been linked to both drought and heat tolerance. Here again, the evidence base is larger and clearer for the major cereal crops than it is for legumes. A study of 56 varieties of chickpea found a difference in CTD between heat tolerant and heat sensitive varieties (Kumar et al., 2017b). On the other hand, a study extending analysis to chickpeas, lentils, and faba beans found that although heat tolerant varieties exhibited lower mean canopy temperatures, differences between heat tolerant and heat sensitive varieties were not statistically significant (Ibrahim, 2011). A single study exists in which leaf temperature is compared between common bean genotypes at high temperatures. No significant difference was reported (Traub et al., 2018).

There are mechanisms underlying interspecies variation in transpirational cooling that are common across crops. Recent work has shown that heat tolerance in wheat is associated with root architecture. Under drought stress, genotypes that were better at canopy cooling had deeper roots, while under heat stress, the same genotypes displayed greater concentration of shallow roots, maximising access to water (Pinto and Reynolds, 2015). Pinto and Reynolds (2015) were subsequently able to identify Quantitative Trait Loci (QTL) for root behavior, providing a common genetic basis for canopy cooling in wheat genotypes. QTL for canopy cooling have also been identified in rice plants. Here, the genetic control for cooler canopies operates through deeper rooting and increased stomatal conductance. Interestingly, this QTL did not significantly correlate with QTLs for drought tolerance, indicating that improvements in yield from canopy cooling can also be conferred directly through stomatal conductance and photosynthesis (Fukuda et al., 2018). In chickpea, molecular markers were able to explain a significant share of variance in CTD and were linked to drought tolerance. Four out of five drought tolerant varieties shared these molecular markers, suggesting that high throughput phenotyping of deeper rooting varieties with cooler canopies may be viable (Purushothaman et al., 2015). Connections between CTD, deeper rooting behavior and drought tolerance have also been found in common bean (Polania et al., 2016). Associated QTLs have not yet been discovered, and we do not know if deeper rooting behavior is associated with heat tolerance in common bean.

Crops also share a second mechanism connecting intraspecies variation in CTD. Intraspecies variation in stomatal response to vapor pressure deficit (VPD) has been found across crops in both controlled and field conditions (Sinclair et al., 2017). Water saving genotypes respond to high temperature and high VPD by decreasing stomatal conductance and conserving water for later in the season. These drought tolerant varieties therefore exhibit a lower transpiration rate in high temperature and high VPD conditions. Water saving genotypes are often more drought tolerant than their water spending counterparts. Transpiration limiting behavior is temperature sensitive (Sinclair et al., 2017). At higher temperatures, some varieties lose their transpiration limiting response to changes in VPD. This modulation of stomatal conductance by environmental conditions suggests that there may be a dynamic trade-off between drought tolerance and heat tolerance, where transpiration limiting traits control interspecies variation in CTD (Tardieu, 2011). Breeding for heat tolerance *via* enhanced cooling therefore requires careful analysis of target population of environments (TPEs) (Tardieu, 2011).

The literature demonstrates that transpirational cooling is an important mechanism for heat avoidance in food crops. It also shows that there is robust evidence for both inter and intra species variation in transpirational cooling, and that there are common mechanisms across crops that determine genotypic variation in this trait. Furthermore, we do not yet know if transpirational cooling is an important mechanism for heat avoidance in common bean nor whether this trait is linked to heat tolerance.

Our first objective is to test whether (i) the magnitude and range of transpirational cooling is sufficient to reduce heat stress. Our second objective will be to test whether (ii) transpirational cooling varies with heat tolerance. Answering this question will help breeders determine whether it is worth breeding for cooler beans. Our third objective will test whether (iii) the association between leaf cooling and VPD varies with heat tolerance. A larger association between leaf cooling and VPD would be indicative of a greater transpirational response to the atmospheric demand for water. Finally, assessing the value of enhanced leaf cooling requires genotype specific modeling of leaf temperature under a range of environments. We therefore build a model estimating leaf temperature from meteorology. Our fourth and fifth objectives will be to test whether (iv) leaf temperature can be modeled using meteorology under well-watered conditions and (v) if leaf temperature-meteorology interactions are genotype dependent. Genotype specific modeling of leaf temperature will allow breeders to assess the value of greater leaf cooling as a criterion for selection. It will help crop modelers to assess the need/feasibility of genotype specific modeling of leaf temperature in heat stress assessments.

## Materials and Methods

### Study Site

**Figure 1:**
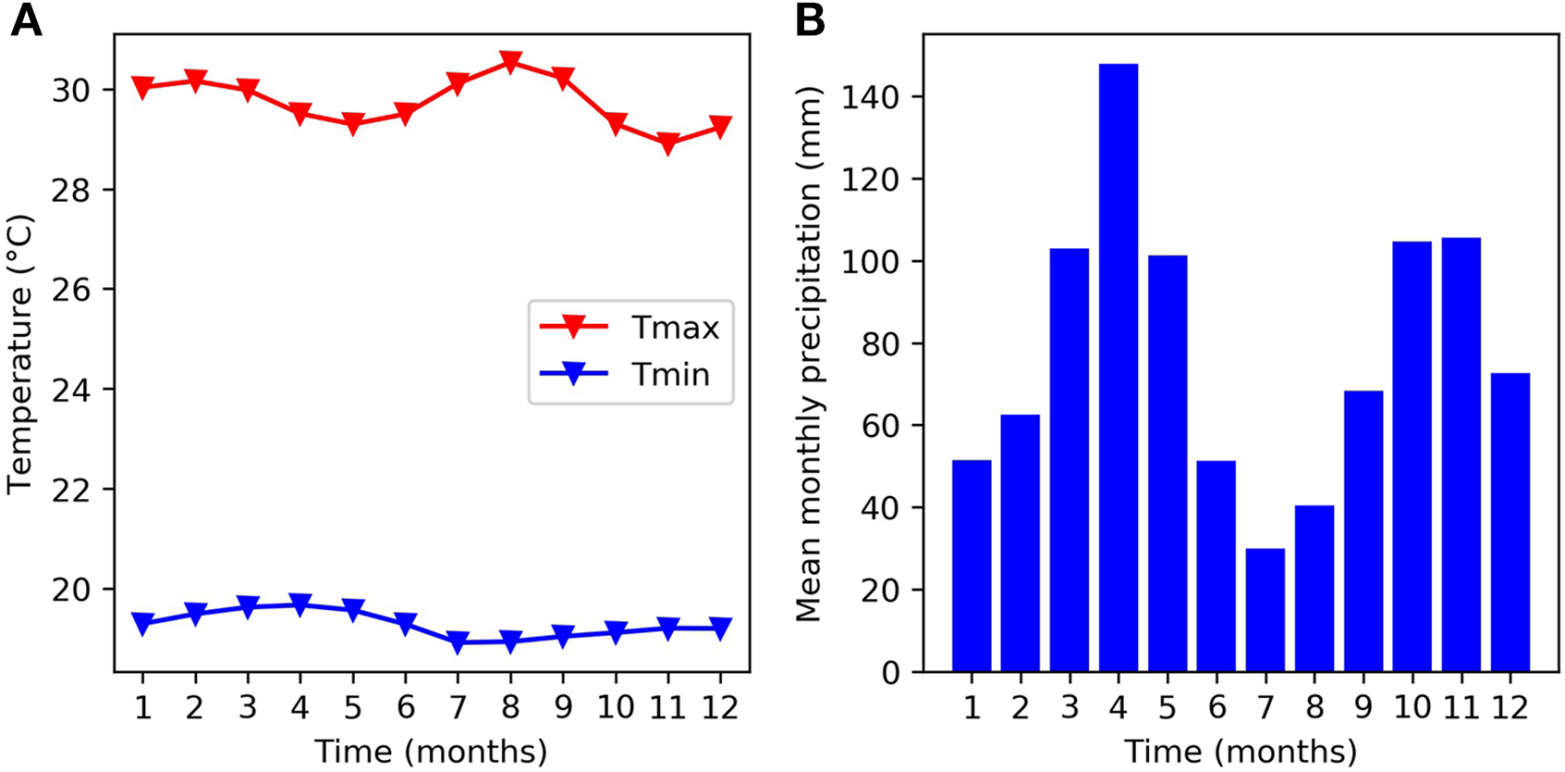
Mean monthly climatology at the experimental site (CIAT HQ) between 1978 and 2018 for **(A)** temperature and **(B)** rainfall.

The experiments used in this study took place at the headquarters of The International Center for Tropical Agriculture (CIAT) in Cali, Colombia - 965 m above sea level (3°29” N, 76°21” W). **Figure 1** provides a monthly climatology of temperature and precipitation for the CIAT HQ between the years 1978 and 2018. The mean monthly maximum temperature remains close to 30°C and the mean monthly minimum temperature remains close to 18°C over the course of the year. These temperatures are just below the thresholds at which bean crops are expected to experience some daytime and night time heat stress (Porch et al., 2010). There are two rainy seasons during the year, which correspond to the two main bean growing seasons. The main rainy season takes place in March, April, and May and a second rainy season takes place in October, November, and December. The soil is a Mollisol (fine-silty mixed, isohyperthermic Aquic Hapludoll) as described by the USDA classification system, with no major fertility problems (pH = 7.7). For a more detailed description of the experimental site, see Beebe et al. (2008) and Rao et al. (2017).

### The Experiments

The data used in this paper is taken from six experiments, each organized in randomized complete block design. A detailed summary of the experiments used for leaf temperature measurements is available in **Supplementary Material Table 1**. H1 (Urban and Ricaurte, 2018a) and H3 (Urban et al., 2018b) (Urban and van Dam, 2018) consisted of three treatments; an ambient treatment undertaken in field conditions (AMB), a greenhouse control experiment with nighttime temperatures kept at 20°C (GH1) and a greenhouse night heat experiment with nighttime temperatures raised to 24°C (GH2). Throughout this paper, we define ambient to mean grown under field conditions and not subjected to stress treatments. For experiment H3, we only include observations of plants grown in the soil, so that observations are fully comparable with the other experiments. H2 (del mar Angel, 2017) consisted of an ambient treatment undertaken in field conditions (AMB) and a greenhouse night heat experiment with nighttime temperatures kept at 25°C (GH). H2 included measurements of fully developed old leaves (base), fully developed young leaves (upper—if not otherwise specified, this is the stage normally taken for all measurements) and young leaves that were not yet fully developed (top). In each of the greenhouse experiments, there was some evidence to suggest that the bean plants may also have experienced stress from above optimal soil pH (pH = 8.1). All of these treatments were kept well-watered.

The drought stress experiment (D1) (Urban et al., 2018a) involved three treatments; an ambient treatment undertaken in well-watered field conditions using drip irrigation, and two water limited treatments grown under a rain shelter using sprinkler irrigation. In the first of these treatments (the early drought treatment), watering ceased 27 days after sowing for a period of 15 days, after which it was kept at 80% of field capacity. In the second treatment, watering ceased 30 days after flowering. The rain shelter remained open when it was not raining. The soil experiment (S1) (Urban and Ricaurte, 2018b) consisted of a single treatment. Six genotypes were cultivated on compacted soils following a recent rice growing season. Plants were kept fully irrigated throughout the season. We used a second drought stress experiment (D2) to compare Specific Leaf Area (SLA). This was part of a bigger experiment called BASE 100 (Bean FOR Abiotic Stress Evaluation, 100 genotypes). The experiment consisted of two treatments, control (nine irrigations) and drought (four irrigations), with the final irrigation 30 days after sowing. Both treatments were conducted in experimental fields at CIAT. SLA was measured 38/39 days after planting (DAP) and 58/60 DAP in both treatments. For each of these days, our measurements contain 15 leaves per genotype (three repetitions of five leaves). For each repetition, the trifoliar leaf was cut off so that the central and side leaves could be measured separately. After the leaf area was measured, each repetition was dried at 70°C for 3 to 4 days until constant weight was achieved. The five central leaves were weighed together and the 10 side leaves were weighed together.

### Instrumentation

We collected observations of air temperature, leaf temperature, relative humidity, leaf thickness, and leaf angle using the MultispeQ v1 device made by PhotosynQ. MultispeQ v1 is a handheld device with a PAR sensor on top of the device and temperature and humidity sensors on the right of the leaf clamp. A small infrared thermometer is housed in the bottom of the device. The precision of ambient temperature, leaf temperature, and relative humidity sensors is 0.5°C, 0.1°C, and 3%, respectively (PhotosynQ, 2019). The device uses photodiodes placed above and below the leaf clamp to measure absorbance at 450, 535, 605, 650, 730, 850, and 940 nanometres. These measurements are used to derive a variety of absorbance and fluorescence-based indicators of photosynthetic activity and leaf health (Kuhlgert et al., 2016). The protocol for leaf measurement is as follows: to take a measurement, we positioned ourselves to avoid casting a shadow over either the leaf or the PAR sensor and placed the MultispeQ device over a central portion of a fully developed young leaf without altering leaf angle. While the leaf was held in the MultispeQ device, the infrared thermometer made a contactless observation of leaf temperature in less than 1 second. At no point did the IR sensor touch the leaf. Throughout a measurement, two vents in the leaf clamp maintained air exchange. The working device protocol was called Photosynthesis RIDES no open/close.

We took measurements of stomatal conductance during experiment H2 using the SC-1 Leaf Porometer from METER group. We always measured the central axial part of the leaf, where most stomata in bean are located. We took measurements of the youngest fully developed leaf (upper) and the youngest not fully developed leaf (top). Instrument preparation, calibration, and measurements were performed as recommended by the manufacturer (Metergroup, 2019). The device has a range of 1–1,000 mmol/m^2^s, a resolution of 0.1 mmol/m^2^s, and an accuracy of 10% from 0 to 500 mmol/m^2^s. Beyond this range, the device is able to measure relative change in stomatal conductance, but the manufacturers are not able to verify the absolute accuracy of the device. The operating temperature of the device is 5–40°C and the operating relative humidity is 1%–100%. We took 182 measurements within this range over 5 days. We took 92 measurements from the ambient treatment and 90 from the greenhouse treatment. In total, we obtained 95 successful measurements for Calima and 87 for SAB686. Measurements were taken over the course of the day at 8 am, 10 am, 1 pm, and 3 pm.

During experiment D2, leaf area measurements were made using the Licor LI-3100C meter for the harvest taken 38/39 DAP from the control experiment. Leaf area measurements made from all other harvests were made with the LI-3000C LA meter connected to an LI-3050 transparent conveyor accessory from the same manufacturer. The resolution of all LA measurements was 1mm squared, with an accuracy of 2% (LICOR, 2019).

### Data Selection

We tested objective (i) using the aggregated data from the five experiments (called the whole sample from here onwards) and for a subset containing only observations taken under ambient conditions (called the ambient subset from here onwards). **Table 1** shows that the mean air temperature is similar in the whole sample and the ambient subset. The standard deviation and range of temperatures is 0.5°C and 3.6°C larger in the whole sample. The difference between samples is larger for relative humidity than for air temperature. The mean, standard deviation and range of relative humidity is lower in the ambient subset than in the whole sample.

**Table 1:** Summary statistics for MultispeQ samples calculated from the whole sample and ambient observations only.

Data	Variable	Mean	Std	Min	Max	Range
Whole sample	Air temperature (°C)	33.6	3.3	26.4	45.0	18.5
Ambient only	Air temperature (°C)	34.1	2.8	26.4	41.4	14.9
Whole sample	Relative Humidity (%)	59.2	8.7	32.7	75.8	43.1
Ambient only	Relative Humidity (%)	55.5	7.8	34.4	75.4	41.0

**Figure 2:**
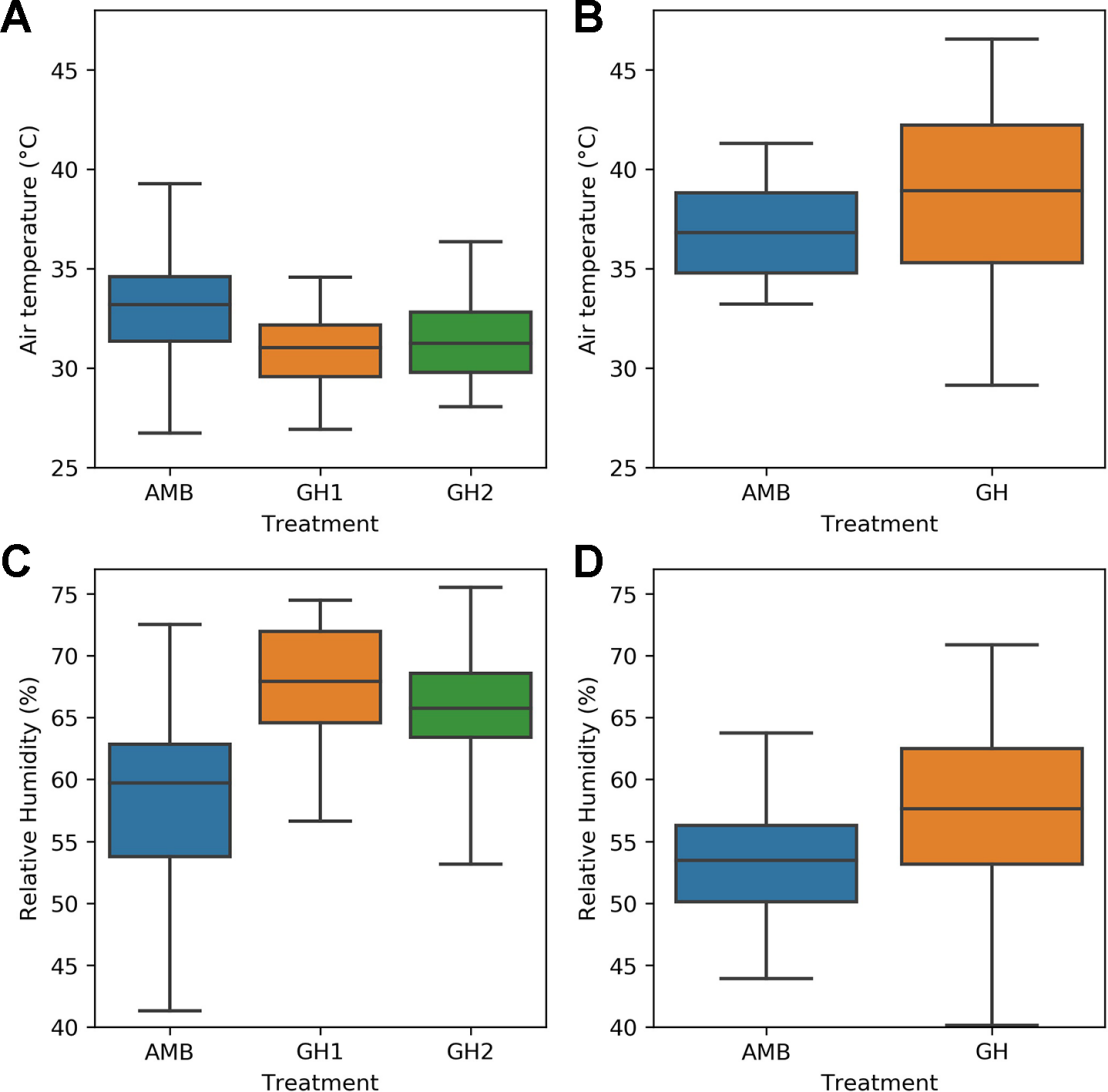
Comparison of sampled air temperature and relative humidity in the H1 and H2 experiments: **(A)** temperature in the H1 experiment **(B)** temperature in the H2 experiment **(C)** relative humidity in the H1 experiment **(D)** relative humidity in the H2 experiment.

The remaining objectives required a comparison of heat sensitive and heat tolerant genotypes. We therefore tested them on the H1 and H2 experiments, because the same heat sensitive and heat tolerant genotypes were used in both experiments and the number of measurements taken were sufficient for statistical analysis. **Figure 2** compares the MultispeQ measurements taken in the H1 and the H2 experiments. **Figure 2** demonstrates that the measured temperatures in the H2 experiment were hotter than the measured temperatures in the H1 experiment. In both of the H2 treatments, the median sampled temperature was above 37°C and the upper quartile of temperatures would be expected to impose heat stress on common bean plants. **Figure 2** also shows that relative humidity was lower in the H2 measurements than in the H1 measurements.

**Table 2** gives a detailed comparison of air temperature, relative humidity, and PAR during the daytime in each of the three H1 treatments. The mean air temperature was similar in all of the treatments. The standard deviation was higher in the ambient treatment than in either of the two greenhouse treatments, meaning that plants experienced more variable daytime air temperatures in the ambient treatment. The minimum and maximum temperatures were lowest in the ambient treatment and highest in the night heat treatment. Mean relative humidity was lowest in the ambient treatment and similar in both greenhouse treatments. Relative humidity was more variable in the ambient treatment and similar in both of the greenhouse treatments. Mean photosynthetically active radiation (PAR) was much larger in the ambient treatment than in the two greenhouse treatments. The standard deviation and maximum of PAR was also higher in the ambient treatment, as expected.

**Table 2:** Summary statistics for each of the treatments in the H1 experiment. Calculated from daytime observations from an in-situ weather station.

**Data**	**Variable**	**Mean**	**Std**	**Min**	**Max**	**Range**
H1 Ambient	Air temperature (°C)	26.6	3.7	17.1	34.7	17.6
H1 GH Control	Air temperature (°C)	25.8	3.3	18.4	36.4	18
H1 GH Night Heat	Air temperature (°C)	26.3	2.7	19.4	39.9	20.5
H1 Ambient	Relative Humidity (%)	58.1	19.6	19.6	100	80.4
H1 GH Control	Relative Humidity (%)	70.5	13.9	33.3	99.7	66.4
H1 GH Night Heat	Relative Humidity (%)	68.7	13.5	30.3	100	69.7
H1 Ambient	PAR (µmol/m2s)	811.1	624.1	9	2,457	2,448
H1 GH Control	PAR (µmol/m2s)	449.1	343.3	9	1,613	1,604
H1 GH Night Heat	PAR (µmol/m2s)	465.0	349.2	9	1,610	1,601

In the H1 experiment, plants were sampled between 8:30 am and 10:45 am in the morning and 1:30 pm and 3:15 pm in the afternoon. In the H2 experiment, plants were sampled between 8 am and 9 am, between 10 am and 11 am, between 1 pm and 2 pm, and between 3 pm and 4 pm. We are therefore only able to capture the impacts of high daytime temperatures as night time temperatures were not sampled. We are therefore able to capture the impact of high daytime temperatures on leaf temperature depression, but not those of high night time temperatures.

### Data Preparation

The MultispeQ device automatically flags potentially unreliable measurements by including a binary issues variable. The device automatically flags measurements during which it was not held steady or if the leaf did not fully cover the light guide. It can also issue a warning flag if measurements of the realized steady state efficiency of photosystem II (ØII), the quantum yields of non-photochemical exciton quenching (ØNPQ), and non-regulatory energy dissipation (ØNO) values are outside the expected range. In our data set, flagged measurements largely referred to instances where the device was not held steadily. In this analysis, we removed all measurements with an issues flag and all measurements which contained missing data for any of the variables. Our analysis therefore only contains complete measurements for all variables without potential issues. Employing this protocol results in the loss of approximately 3.7% of the total samples taken. For genotype comparisons, in which we made use of variables with a higher propensity of measurement error, we further removed measurements that were more than three times the interquartile range above the third quartile and below the first quartile.

The arithmetic mean of technical replicates for each genotype are treated as independent random samples for the purposes of testing differences between genotypes. Only sampling days when both genotypes are tested during the same time periods are included in the analysis. Three replicates were taken in the H1 experiment. Post data preparation and averaging of replicates, the H1 experiment consisted of 821 independent observations, 258 observations from the ambient experiment, 271 observations from the greenhouse control experiment, and 292 observations from the greenhouse night heat experiment. Three replicates were taken in the H2 experiment. Post data preparation and averaging of replicates, the H2 experiment consists of 318 independent observations, 96 from the ambient treatment, and 222 from the greenhouse night heat treatment.

### Plant Material

Three contrasting genotypes were grown in the H1 experiment. Calima is a heat/drought sensitive check variety, grown throughout Colombia, SAB 686 is a heat/drought tolerant variety and SEF 60 is a heat tolerant variety. Both Calima and SAB 686 are common bean varieties from the Andean gene pool. Calima produces medium sized seeds with a red mottled colour and SAB 686 produces medium sized seeds of a cream mottled colour. Both genotypes are growth types 1; strong and erect systems. SEF 60 is a triple interspecific cross with Tepary bean (*P. acutifolius*) and Runner bean (*P. coccineus*). Tepary bean originated in arid and semi-arid conditions (Mhlaba et al., 2018) and has been shown to enhance heat tolerance when crossed with common bean varieties (CIAT, 2015). SEF 60 produces medium sized red seeds and is resistant to Bean Common Mosaic Necrosis Virus (BCMNV). SEF 60 is from the Mesoamerican gene pool, with growth type 2A; indeterminate erect systems without guidance. Calima and SAB 686 were also grown in the H2 experiment.

### Variable Definitions

The term CTD is often used interchangeably to describe the difference between canopy and air temperature and the difference between leaf and air temperature. In this paper, we will use the term leaf temperature depression to make clear that we are referring specifically to the difference between air and leaf temperatures. The leaf temperature depression is a good indicator of the CTD at the top of canopy. Leaf temperature depression (LTD) was calculated from the air temperature and the leaf temperature measured by the MultispeQ device.

(1) LTD=Leaf temperature−Air temperature

VPD was calculated by subtracting the actual vapor pressure (ea) from the saturated vapor pressure (es). We used the Magnus method (Andersson-Sköld et al., 2008) for calculating the saturated vapor pressure.

(2) $$es=0.61094\;*e^{\left(17.625T/T+243.04\right)}$$

The actual vapor pressure was then calculated using the relative humidity (RH) as follows.

(3) ea=RH/(100es)

The VPD is then given by

(4) VPD=es−ea

### Statistical Methods and Inference

In this paper, we conducted hypothesis tests that rely on statistical comparison of group means. We tested the hypothesis that heat tolerant genotypes are cooler than heat sensitive genotypes. Since LTD is a negative number, this equates to the following hypothesis test:

Ho: The mean LTD of the heat sensitive genotype is identical to the mean LTD of the heat tolerant genotypes.

Ha: The mean LTD of the heat sensitive genotype is greater than the mean LTD of the heat tolerant genotypes.

We used a one-sided permutations test (Ludbrook and Dudley, 1998) to conduct the above hypothesis test. For our application, we chose a permutations test instead of a nonparametric Mann-Whitney U test, as there is no theoretical justification for assuming that the distribution of LTD is the same shape for each genotype. If this assumption were violated, then the Mann-Whitney test would not be a comparison of the averages of the two groups of data.

Permutation testing for equal means between two observed samples begins by concatenating these samples. The concatenated array is then randomly shuffled. This shuffled array (known as a permuted sample) is split into two separate arrays of the same length as the two input samples. The difference in means between these two arrays is then calculated. We repeated this process 10,000 times, resulting in 10,000 permuted samples and mean differences. The p-value was then computed by calculating the proportion of permuted samples in which the mean difference was greater than the mean difference in the observed samples. This provided an estimate of the probability of the difference in means being larger than the observed difference in means by chance.

In addition to testing for differences in the mean LTD between heat tolerant and heat sensitive genotypes, we are also interested in how cooling responds across the temperature distribution. Since there is no theoretical reason to suppose a linear relationship between LTD and temperature over the whole temperature distribution, we used a local regression to examine this assumption. Local regression fits a linear or quadratic function to a moving window of the input data set (Cleveland and Devlin, 1979). This window is the locality described in the name local regression. The size of the locality (the proportion of the data set used in each window) is user defined and determines how smooth the fit produced is (Cleveland and Devlin, 1979). We used two-thirds of the data in each moving window. Observations used in the regression were weighted by their distance from the observation being fitted. We used a bisquare function of the residuals to weight each observation three times. We performed and plotted the lowess regression using Python's Seaborn library. The smoothed results of this locally weighted regression are presented on a scatter plot, which we describe as a lowess regression in the results section. The purpose of this exercise was to visually examine the form of LTD across the temperature distribution.

We tested objective 3 (that the relationship between LTD and VPD varies with the tolerance of the genotype) by using a Spearman rank correlation (Zwillinger and Kokoska, 1990) from Python's SciPy library. The relationship between LTD and VPD is noisy and nonlinear, since as discussed in the introduction, LTD is also controlled by leaf traits and other environmental variables. This is the reason that we chose to use a rank correlation instead of a Pearson correlation.

Objective 4 requires the development of a model to predict leaf temperature using meteorological conditions. We combined the data from the H1 and H2 experiments to ensure that our model performs well in a range of temperatures and relative humidity. We subset the H2 data to only include samples taken from the upper leaf in the canopy to ensure comparability between the two data sets. Since we wanted the model to only use variables that are available to crop modelers, we built on success in predicting rice canopy temperatures using air temperature and relative humidity (Van Oort et al., 2014). We expected the impact of air temperature on leaf temperature to vary at different levels of relative humidity, so we included an interaction term. Finally, we included a dummy genotype variable to test for impact on model performance.

Environmental variables are often highly correlated with each other and strong correlation between temperature and relative humidity introduces a multicollinearity problem for Ordinary Least Squares (OLS) regression. We therefore used a ridge regression, a variation of OLS that increases the stability of the regression coefficients by renegotiating the bias vs. variance trade-off in favor of reducing variance. Ridge regression is an effective way of reducing the impacts of multicollinearity on regression coefficients and is applied to scaled independent variables (Sen and Srivastava, 1990). Scaling was performed by subtracting the mean and dividing by the standard deviation to ensure that the mean of each input variable is equal to 0 and the standard deviation is equal to 1.

(5) $$Scaled\left(X_{i}\right)=\left(X_{i}-mean\left(X\right)\right)/standarddeviation\left(X\right)$$

We present the form of the regression below

(6) $$\hat{y}=\beta_{0}+\beta_{1}sctair+\beta_{2}scrh+\beta_{3}sc\left(tair*rh\right)+\beta_{4}gen+\epsilon$$

sctair = scaled air temperature, scrh = scaled relative humidity, sc(tair*rh) = scaled temperature and relative humidity interaction term and gen = binary genotype variable, which is equal to 1 for Calima and 0 for SAB 686.

Ridge regression selects the regression coefficients based upon a variation of the OLS loss function. An additional term is added to the loss function comprising the squared value of the regression coefficients. This effectively penalizes the selection of large coefficients. The formal description of selection of coefficients in a ridge regression is given below in equation 7

(7) $${\hat{\beta }}_{\left(k\right)}={\left(X^{\prime }X+kI\right)}^{-1}X^{\prime }Y$$

where Y is the observations, X is the independent variables, and I is the Identity matrix (Ryan, 1997). Note that when k = 0, the ridge regression collapses to an OLS regression.

Before applying the ridge regression, we randomly split the data into 70% training data and 30% testing data. We used the Train-Test-Split function in Python's sklearn library with seed = 1 to perform this random split, employing stratification by experiment, treatment, and genotype to ensure a balanced sample. The training data was used to fit the regression and the testing data was used to evaluate the regression. Measures of model performance reported in this paper are based on the performance of the regression on the testing data alone. The selection of k in equation 7 was performed using a grid search of values between 0 and 1 with a search resolution of 0.1. The criterion for selection of k was maximizing r-squared and we tested each value of k in the grid using fivefold cross-validation on the training data set. We used a Scikit learn pipeline to perform both regression training and grid search operations.

## Results

### LTD is an Important Heat Avoidance Mechanism

Leaf temperature depression was large in our study, demonstrating that leaf cooling strongly regulated leaf temperature (**Table 3**). On average, the temperature of the leaf was 5.2°C cooler than the temperature of the air and varied between 13°C cooler and 2.1°C warmer.

**Table 3:** Summary statistics of MultispeQ observations of air temperature, leaf temperature and leaf temperature depression(°C). Calculated from the whole sample.

Variable	Mean	Std	Min	Max	Range
Air temperature	33.6	3.3	26.4	45.0	18.5
Leaf temperature	28.4	3.1	21.9	42.0	20.1
Leaf temperature depression	−5.2	1.9	−13.0	2.1	15.1

**Figure 3:**
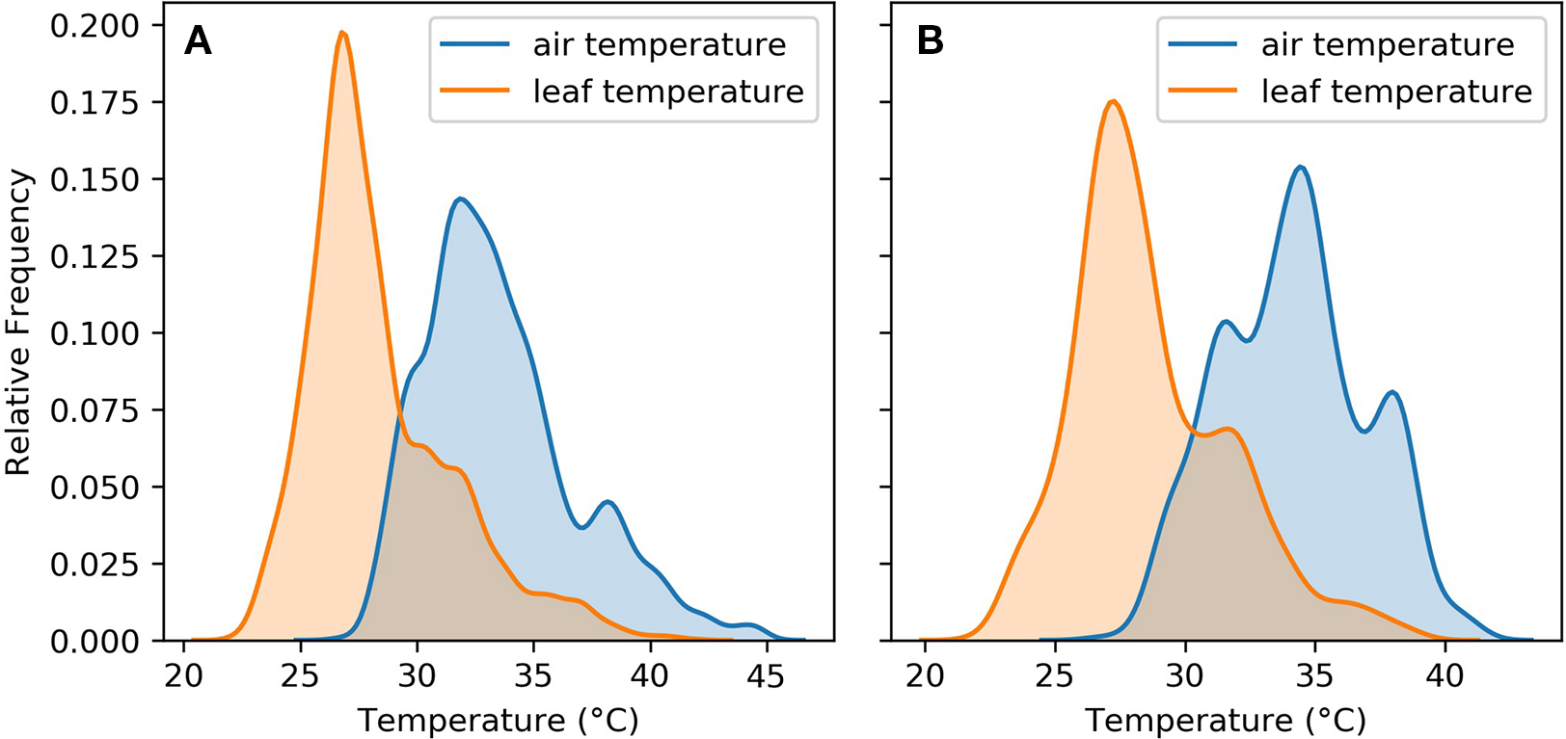
Air and leaf temperature distributions for **(A)** aggregated observations from the five experiments **(B)** aggregated observations from the five experiments in ambient treatments only.

**Figure 3** shows that leaf cooling played an important role in keeping leaf temperatures within the range required to maintain their physiological function. This was the case for both the whole sample and the ambient subset. In both the whole sample and the subset of ambient observations, the peak of the leaf temperature distribution was within 25°C–30°C.

### LTD Varies With Heat Tolerance

**Figure 4:**
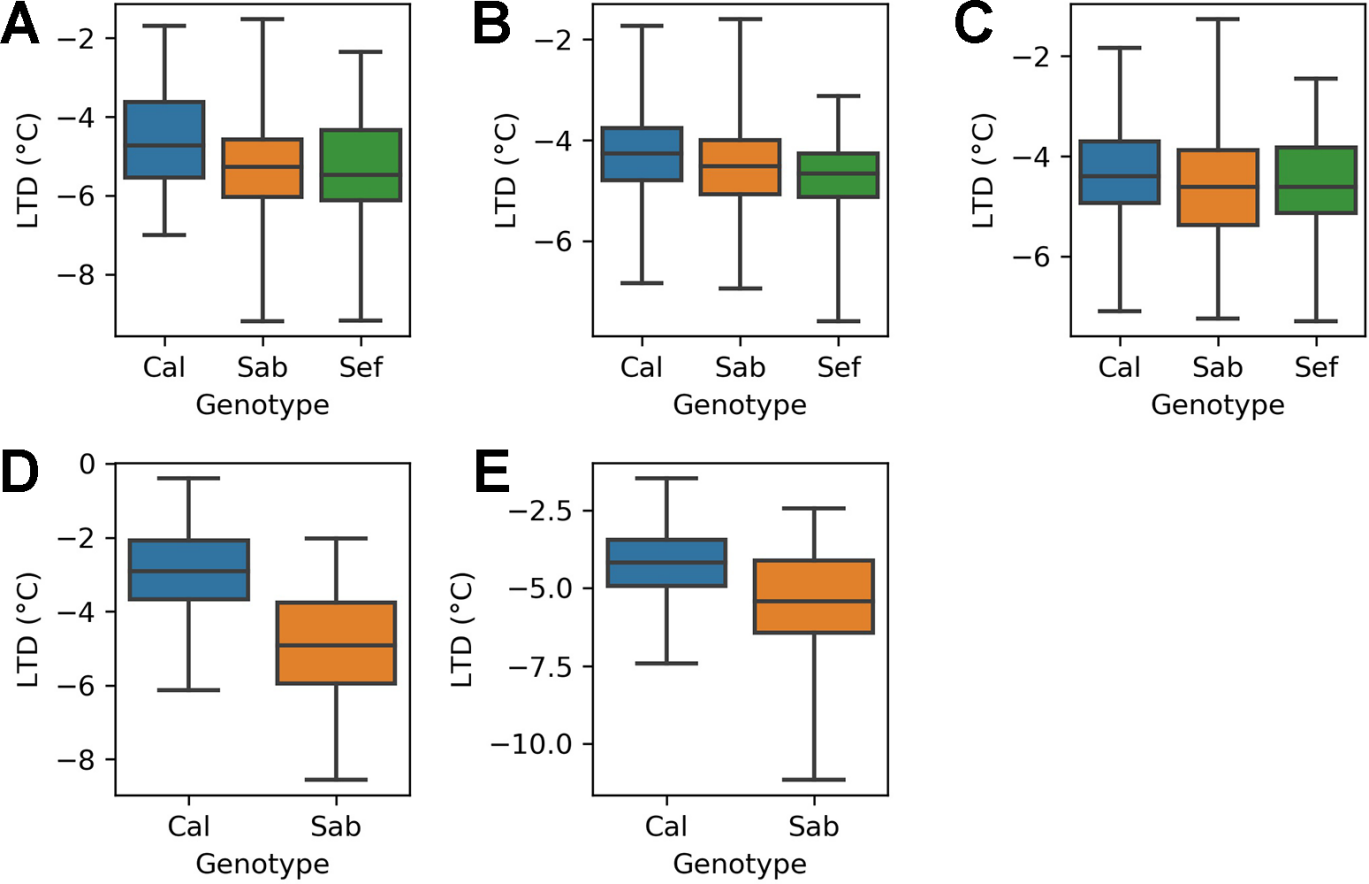
Distribution of leaf temperature depression (LTD) observations by treatment and genotype for the H1 and H2 experiments: **(A)** H1 experiment - ambient treatment **(B)** H1 experiment - GH control treatment **(C)** H1 experiment - GH night heat treatment **(D)** H2 experiment - ambient treatment **(E)** H2 experiment - GH night heat treatment .

The heat tolerant varieties cooled by more than the heat sensitive variety in all three treatments of the H1 experiment (**Figure 4**). In the ambient treatment (A), the heat tolerant varieties (SAB 686 and SEF 60) cooled 0.77°C and 0.82°C more than the heat sensitive variety (Calima). These differences are statistically significant at the 95% confidence level (**Table 4**). In the Greenhouse control treatment (B) SAB 686 cooled 0.2°C more than Calima, this difference is not statistically significant at the 95% confidence level (**Table 4**). SEF 60 cooled 0.5°C more than Calima, this difference is statistically significant at the 95% confidence level (**Table 4**). In the Greenhouse night heat treatment (C) SAB 686 cooled 0.2°C more than Calima and SEF 60 cooled 0.1°C more than Calima. Neither of these differences are statistically significant at the 95% confidence level (**Table 4**).

**Table 4:** Test statistics for a comparison of leaf temperature depression (LTD) group means between genotypes for each treatment of the H1 and H2 experiments.

Experiment	Test	Treatment	Test-statistic	p-value
H1	Calima vs. SAB 686	Ambient	permutation test	0.00
H1	Calima vs. SEF 60	Ambient	permutation test	0.00
H1	Calima vs. SAB 686	Greenhouse control	permutation test	0.11
H1	Calima vs. SEF 60	Greenhouse control	permutation test	0.00
H1	Calima vs. SAB 686	Greenhouse night heat	permutation test	0.10
H1	Calima vs. SEF 60	Greenhouse night heat	permutation test	0.25
H2	Calima vs. SAB 686	Ambient	permutation test	0.00
H2	Calima vs. SAB 686	Greenhouse night heat	permutation test	0.00

The H2 experiment supports the hypothesis that SAB 686 cools more than Calima. In the ambient treatment (D), SAB 686 cooled 2°C more than Calima and this difference is statistically significant at the 95% confidence level. In the GH night heat experiment (E), SAB 686 cooled 1.3°C more than Calima and this difference is also statistically significant at the 95% confidence level. In addition to greater mean cooling, SAB 686 also exhibited greater variability and a larger range of leaf cooling.

**Figure 5:**
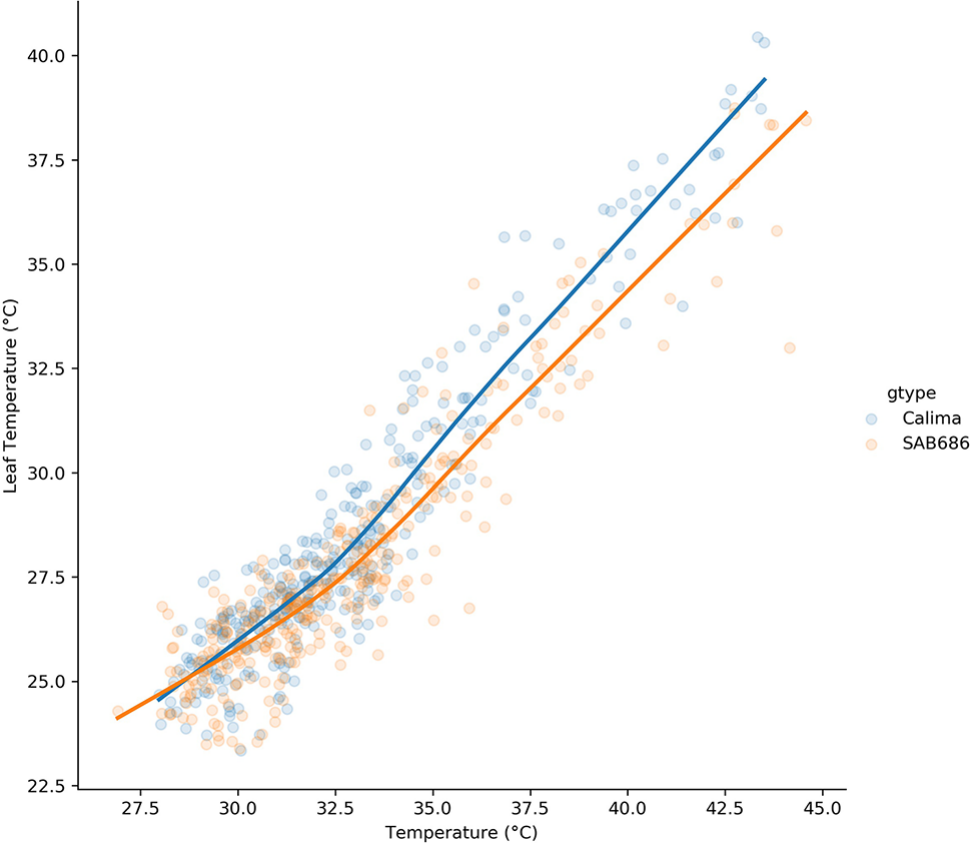
Lowess regression on the pooled data for Calima and SAB 686 from the H1 and H2 experiments.

The lowess regression on the pooled H1, H2 data for Calima and SAB 686 (**Figure 5**) shows that the relationship between air and leaf temperatures was nonlinear for both genotypes. At lower temperatures, the relationship between air and leaf temperatures is similar for both genotypes, however, at higher temperatures, SAB 686 cooled more than Calima leading to a gap in leaf temperatures between the two contrasting genotypes.

### Thermal Gradient Within the Canopy Varies by Genotype

**Figure 6:**
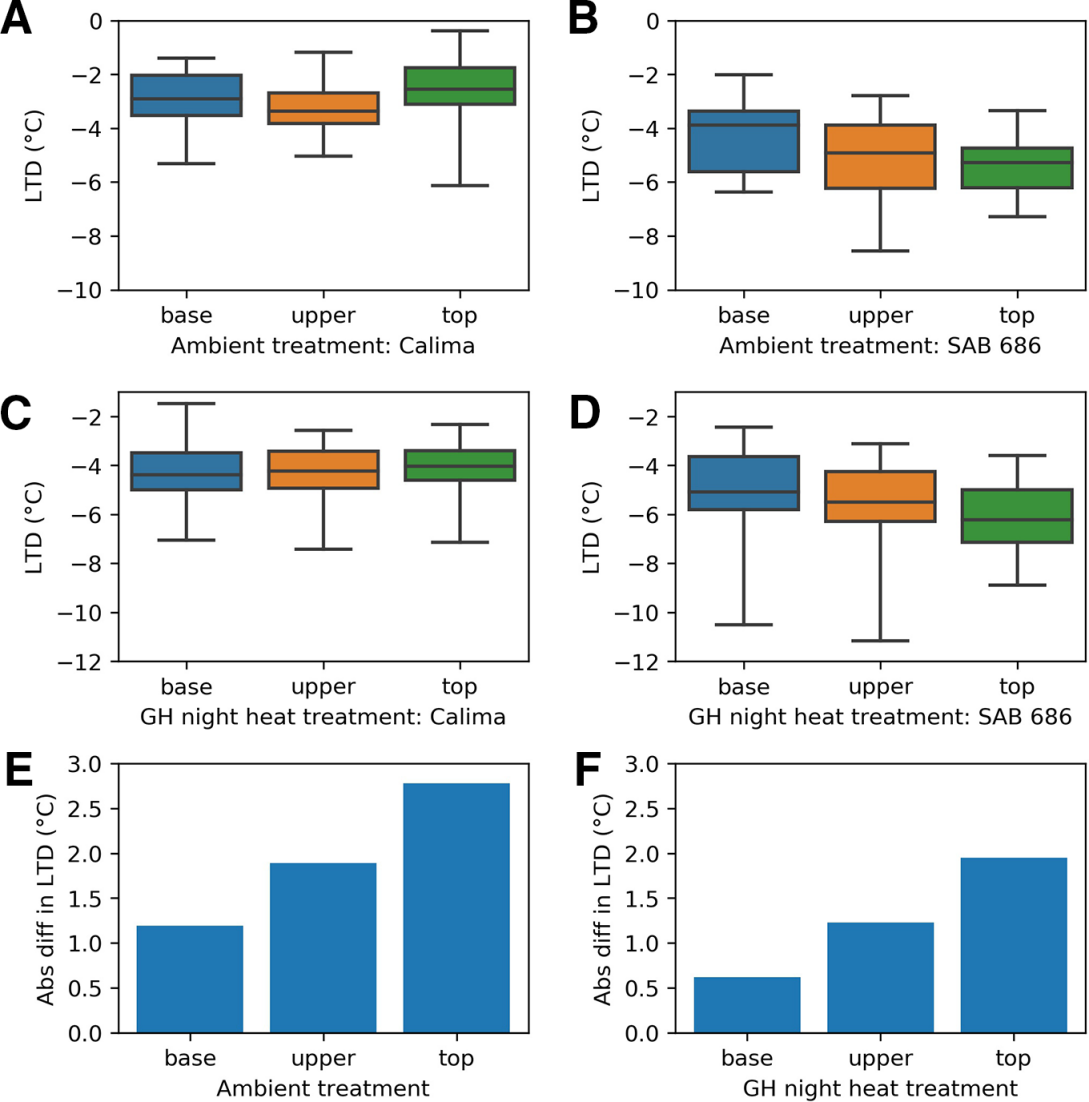
Leaf temperature depression at different positions within the canopy by treatment and genotype in the H2 experiment: **(A)** Calima in ambient conditions **(B)** SAB 686 in ambient conditions **(C)** Calima in night heat conditions **(D)** SAB 686 in night heat conditions **(E)** The absolute difference in leaf temperature depression (LTD) between SAB 686 and Calima in ambient conditions **(F)** The absolute difference in LTD between SAB 686 and Calima in night heat conditions.

For all positions within the canopy and for all treatments of the H2 experiment, SAB 686 cooled more than Calima (**Figure 6**). The gradient in leaf cooling through the canopy differed between the two genotypes (**Figure 6**). In both treatments SAB 686 cooled most at the top of the canopy and least at the bottom of the canopy. Interestingly, this thermal gradient in leaf cooling did not exist for Calima. The difference in the magnitude of leaf cooling between SAB 686 and Calima was greatest at the top of the canopy and smallest at the bottom of the canopy. The last row of **Figure 6** shows that in ambient conditions, SAB 686 cooled 2.8°C more than Calima at the top of the canopy compared with 1.2°C more at the bottom of the canopy.

### There is Genotypic Variation in the Relationship Between LTD and VPD

**Figure 7:**
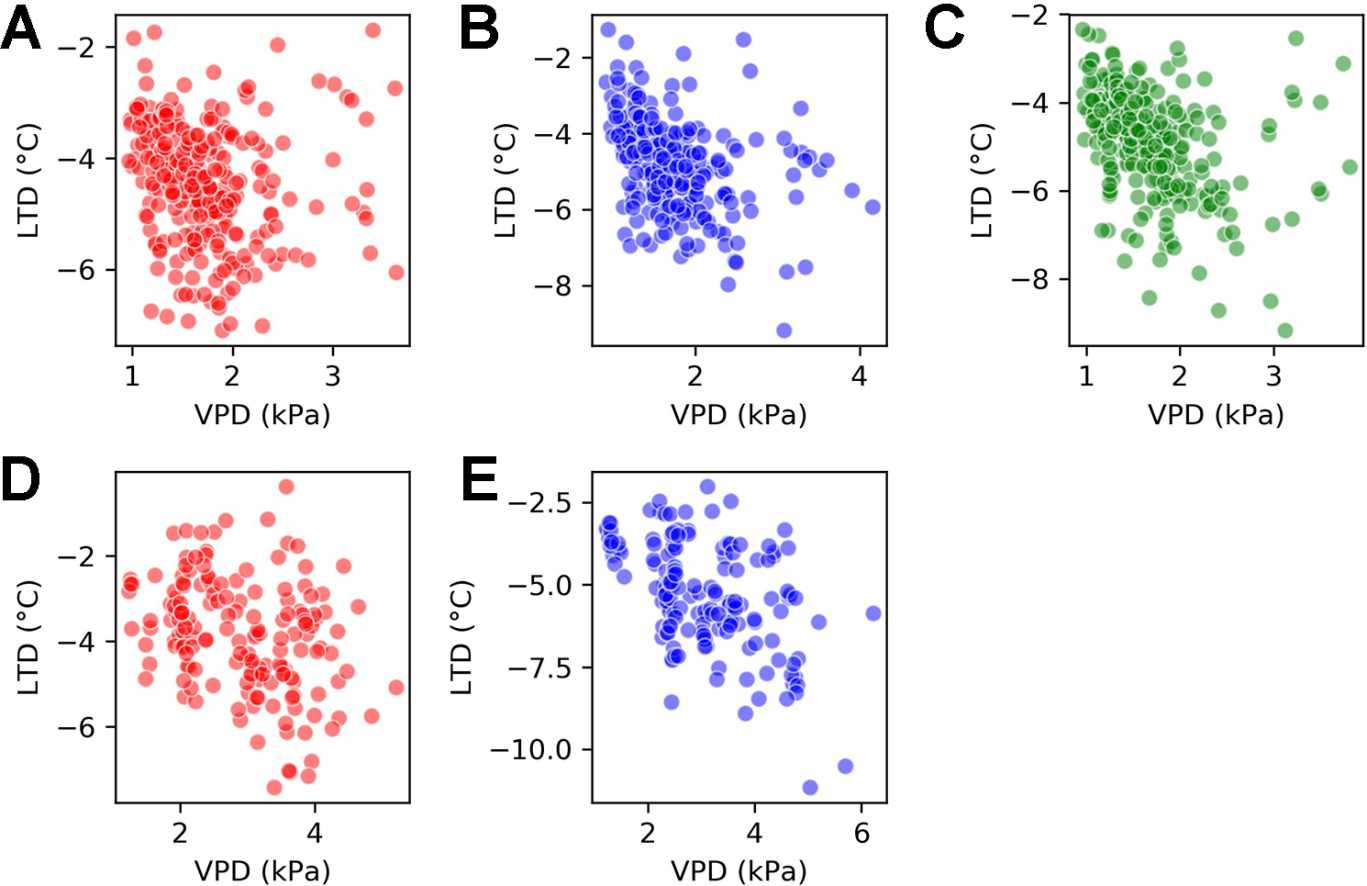
Scatter plots for vapor pressure deficit (VPD) and leaf temperature depression (LTD) by genotype for the H1 experiment and the H2 experiment. **(A)** H1 experiment - Calima **(B)** H1 experiment - SAB 686 **(C)** H1 experiment - SEF 60 **(D)** H2 experiment - Calima **(E)** H2 experiment - SAB 686.

The relationship between LTD and VPD varied by genotype. **Figure 7** shows scatter plots of the joint LTD-VPD distribution for each of the genotypes. The first row takes observations from the H1 experiment and compares all three genotypes and the second row takes observations from the H2 experiment and compares Calima and SAB 686.

Beginning with the H1 experiment (first row of **Figure 7**), there was a clearer association between VPD and LTD for SAB 686 (B) and SEF 60 (C) than for Calima (A). This is shown by Spearman correlations of −0.46 and −0.46 compared with −0.26. The association between VPD and LTD remained greater for SAB 686 (E) than Calima (D) in the hotter and dryer H2 Experiment. The Spearman correlation coefficients for the H2 experiment are −0.46 for SAB 686 and −0.32 for Calima. All correlation coefficients discussed in this section are significant at the 99% confidence level.

### Leaf Temperature is Explained by Air Temperature and Relative Humidity

**Figure 8:**
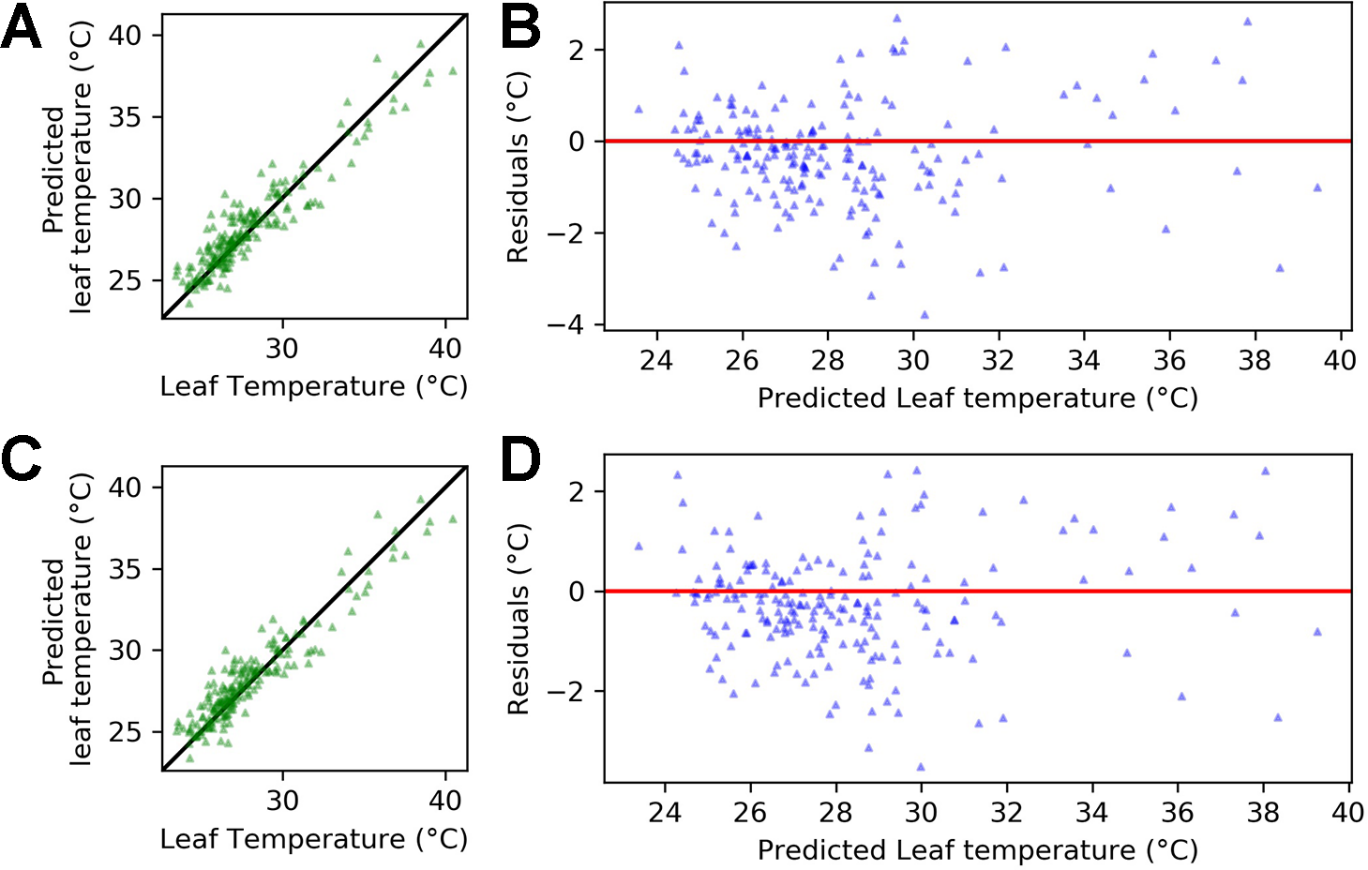
Regression output for equations 8 and 9 applied to the pooled data for Calima and SAB 686 from experiments H1 and H2. **(A)** Leaf temperature vs. predicted leaf temperature - Equation 8 **(B)** Predicted leaf temperature vs. residuals - Equation 8 **(C)** Leaf temperature vs. predicted leaf temperature - Equation 9 **(D)** Predicted leaf temperature vs. residuals - Equation 9. In **(A, C)**, the solid (identity) line represents perfect agreement.

**Figure 8** shows that accuracy in predicting leaf temperature was high using only temperature, relative humidity and an interaction term between the variables (Equation 8). The ridge regression is able to explain 87% of the variance in leaf temperature (r-squared = 0.87) with a root mean squared error (RMSE) of 1.16°C. When including a dummy variable for genotype, the r-squared value increased to 0.88 and the RMSE decreased to 1.11°C (Equation 9).

**Figure 8** plots the predicted leaf temperatures against the error term of the ridge regression. A key assumption required for accurate prediction of regression performance is constant variance of the error term (homoscedasticity). In **Figure 8**, the residuals appear randomly spread around the zero line, which suggests that the homoscedasticity assumption is satisfied.

In both equations 8 and 9, air temperature is the dominant driver of leaf temperature. However, coefficients for relative humidity and the interaction between temperature and relative humidity are also nonzero. This suggests that the impact of temperature on leaf temperature depends on the relative humidity. In Equation 9, the coefficient for the genotype dummy is 0.54. This implies that if we were modeling the heat sensitive variety Calima (gen = 1), then the leaf temperature would be (on average) slightly over half a degree warmer than if we were modeling the heat tolerant variety SAB 686.

(8) $$\hat{LT}=28.3+3.92\left(sctair\right)+1.14\left(scrh\right)-0.75\left(sc\left(tair*rh\right)\right)+\epsilon$$

(9) $$\hat{LT}=28.0+4.05\left(sctair\right)+1.31\left(scrh\right)-0.89(sc(tair*rh))+0.54(gen)+\epsilon$$

## Discussion

### Heat Avoidance Through Transpirational Cooling

In the section *LTD Is an Important Heat Avoidance Mechanism*, we show that leaf cooling shifts the temperature distribution experienced by the upper leaves of the plant to a range in which physiological function is maintained. A second way in which transpirational cooling contributes towards heat tolerance is through maintaining temperatures below damaging biochemical thresholds (Porch and Hall, 2013).

A number of studies have been conducted illustrating the impacts of heat stress on common bean during the reproductive period. Although many pathways to impact have been established by thorough experimental work, different studies have imposed different combinations of day and night time temperatures (Araújo et al., 2015). This makes it hard to pinpoint exactly what daytime temperature threshold results in heat stress. For this reason, we examine the impact of transpirational cooling on a threshold grounded in the biochemistry of photosynthesis.

In C3 plants, photosynthesis declines above a threshold of 35°C as a result of a reduction in the activation state of Rubisco (Salvucci and Crafts-Brandner, 2004; Sage et al., 2008). This limits carbon fixation and subsequently, net photosynthesis. In the whole sample, 27% of air temperature observations were greater than 35°C, while only 4.8% of leaf temperatures were above 35°C. It follows that leaf thermal regulation plays an important role in maintaining photosynthesis at high temperatures in common bean.

### Genotypic Variability in Leaf Cooling

To date, there are many theories seeking to explain the physiological mechanisms through which heat tolerance is conferred in common bean. We asked if heat tolerance could be linked to enhanced leaf cooling. In the section *LTD Varies With Heat Tolerance*, we show that heat tolerant genotypes cool more than heat sensitive genotypes in four out of the five treatments studied. Unlike Traub et al. (2018) we did find significant differences between heat tolerant and heat sensitive genotypes. The size of these differences ranged from 2°C to 0.1°C depending on the environmental conditions.

A difference of 1°C–2°C matters in the context of adaptation to a warming climate. A difference of 1°C–2°C in leaf thermal regulation could conceivably reduce heat damage by reducing heat stress threshold exceedance during extreme temperature events. Differences in leaf cooling of this magnitude could also contribute to heat tolerance by reducing the time the plants spend at sub-optimally high temperatures over the course of the growing season. For example, the plant may cumulatively experience less photorespiration.

In the section *Thermal Gradient Within the Canopy Varies by Genotype*, we show that the difference in the strength of leaf cooling between the heat tolerant variety and the heat sensitive variety is largest at the top of the canopy. Since both genotypes are determinate bush beans and flower from the top to the bottom of the canopy, our results suggest that enhanced cooling in heat tolerant varieties is largest where sensitivity to temperature during the reproductive process is greatest. The combination of the magnitude of enhanced cooling and the place where this enhanced cooling is greatest, suggests an important role for leaf cooling in heat tolerance in common bean. The magnitude of the impact of greater cooling on heat tolerance may also be influenced by the extent of leaf acclimation to heat. Future work should seek to test for interactions between leaf cooling and leaf acclimation.

The results show that the connection between heat tolerant genotypes and greater cooling varies under different combined temperature and relative humidity regimes. The difference in mean cooling between heat tolerant and heat sensitive genotypes was much larger in the H2 experiment, in which mean temperature was higher and mean relative humidity was lower. We also show that the difference in leaf cooling between heat tolerant and heat sensitive genotypes widens at higher temperatures. This suggests that the effectiveness of enhanced cooling as a pathway to heat tolerance may increase as the climate continues to warm.

The evidence suggests that enhanced leaf cooling will be most effective in aiding adaptation in hot and dry conditions. However, given that transpirational cooling relies on water availability, this method of heat avoidance may not be effective in water scarce conditions. Greater transpirational cooling could make these varieties more sensitive to drought if irrigation is not available during dry spells and net transpiration is increased.

### VPD and Leaf Cooling

In the section *There Is Genotypic Variation in the Relationship Between LTD and VPD*, we showed that the association between VPD and LTD does vary with heat tolerance. In both experiments, the heat tolerant varieties cooled more in response to changes in VPD than the heat sensitive genotype. This supports the hypothesis that heat tolerant genotypes exhibit greater transpirational cooling.

A stronger association between VPD and leaf cooling may also confer tolerance by helping to maintain leaf water content. In a series of experiments, Omae et al. (2012) show that heat tolerant snap bean genotypes maintain a higher leaf water content than heat sensitive genotypes under both heat and drought stress conditions. They show that leaf water content is associated with the number of pods per plant and final yield (Omae et al., 2012). In addition, they find that heat tolerant genotypes exhibit a smaller drop in leaf water content at midday and that this difference was associated with a higher pod setting ratio. They propose that an enhanced water potential gradient between the soil and the leaves allows heat tolerant genotypes to absorb more water, preventing dehydration under hot and dry conditions (Omae et al., 2012). Our results support this hypothesis, as a stronger response to VPD in heat tolerant genotypes allows for a stronger water potential gradient.

A stronger cooling response to VPD in heat tolerant genotypes may be the result of higher stomatal conductance. Measurements of stomatal conductance made during experiment H2 (the hotter and dryer experiment) show that the heat tolerant genotype exhibited far higher stomatal conductance during both the hot and dry treatment and the hot and more humid treatment (**Supplementary Material Figure 1**). Our results are in agreement with Tsukaguchi et al. (2003), who also found that heat tolerant snap bean cultivars maintain greater stomatal conductance under high temperature conditions than heat sensitive cultivars (Tsukaguchi et al., 2003). We hypothesize that stomatal conductance is greater in heat tolerant genotypes, which allows for a greater transpirational response to VPD and enhanced transpirational cooling.

It should be noted that greater stomatal conductance leading to greater transpirational cooling will only lead to enhanced leaf water content if water remains available. These characteristics would therefore only contribute to heat tolerance in the presence of a third trait enhancing access to water in heat tolerant genotypes. Candidate traits include deeper root systems (discussed in the introduction), lower root radial hydraulic resistance (higher root conductivity) and greater leaf osmotic adjustment resulting in more stable cell tugor. These are promising avenues of enquiry for future work. If one or more of these hypothesise are true, it would suggest that heat avoidance through transpirational cooling has co-evolved with traits for drought resistance. Given that both of the heat tolerant genotypes we used in these experiments were derived from lines originating in semi/arid environments, co-evolution of traits conferring heat and drought tolerance seems plausible.

Since response to VPD was not reduced in the very hot and dry conditions of H2, our work supports the hypothesis put forward by Sinclair et al. (2017) that transpiration limiting traits are modulated by the plants' environment rather than being attached to absolute transpiration breakpoints (Sinclair et al., 2017). We are in the process of repeating these experiments under varying soil moisture conditions and will explore transpirational cooling and stomatal control in water limiting conditions in future work.

### Leaf Morphology

Differences in LTD are not the result of differences in leaf angle and accompanying differences in incident radiation. In all treatments of experiments H1 and H2, no significant differences in leaf angle between the heat sensitive and heat tolerant genotypes were found (**Supplementary Material Table 2** and **Supplementary Material Figure 2**).

In all three treatments of experiment H1, the heat tolerant genotypes exhibited lower SLA than the heat sensitive genotype. **Supplementary Material Figure 3** shows that in two out of three treatments the difference in mean SLA between the heat tolerant and the heat sensitive varieties was significant at the 95% confidence level. The same pattern was observed in experiment D2 for both ambient and drought conditions (**Supplementary Material Figure 4**). Evidence for differences in leaf area was more mixed. SAB686 had a larger leaf area in ambient conditions, but there was no clear difference in the drought treatment (**Supplementary Material Figure 5**).

A lack of clear distinction in leaf area suggests that differences in SLA was the result of thicker leaves. This is partially supported by **Supplementary Material Figure 6**, which shows MultispeQ measurements of leaf thickness from experiments H1 and H2. In four out of five treatments, the heat tolerant genotypes had thicker leaves than the heat sensitive genotype on average. However, differences were statistically significant at the 95% confidence level in only one out of the five treatments (**Supplementary Material Table 3**). Our sample size was limited in control, drought and high temperature environments. We therefore cannot exclude individual adaptive differentiation processes or plastic responses as reasons for differences in thickness between the two genotypes.

Thicker leaves means a greater thermal mass, which increases thermal stability. Increased thickness can therefore reduce the time spent above damaging temperature thresholds, which explains why leaves are often thicker in hot and dry environments (Leigh et al., 2012). Thicker leaves also provide greater storage space for the accumulation of water within the leaves (the succulent effect), which (very likely) increases thermal stability as well. Leigh et al. (2012) found that small increases in thickness in hot desert conditions with low wind speeds can have a large dampening effect on leaf temperatures. They use a leaf temperature model to demonstrate that this effect is particularly important when hot and dry conditions lead to stomatal closure and transpirational cooling is reduced. Our results suggest that in addition to potential differences in transpirational cooling, the heat tolerant genotypes may have cooled more than the heat sensitive genotype because they had thicker leaves. Differences in leaf thickness were not large enough to prove this, but not weak enough to rule it out. Lower SLA could also be associated with other traits that can increase the thermal resistance of the leaf. For example, leaves with lower SLA may have less permeable leaf cuticles or vary in leaf resistance (glabrous/pubescent leaves may be linked to less/more trichomes and a thin/firm boundary layer).

### Modeling Leaf Temperature

In the section *LTD Is an Important Heat Avoidance Mechanism* we have clearly shown that leaves are consistently cooler than the air and that this difference is large enough to be an important heat avoidance mechanism. We have also shown that there is a G × E interaction in the processes governing leaf temperature. The importance of modeling leaf temperature for assessing genotype value is therefore clear. In the section *Leaf Temperature Is Explained by air Temperature and Relative Humidity*, we show that we can predict upwards of 85% of variation in leaf temperature by genotype in the range of air temperatures covered by these experiments (27°C–45°C).

There are a number of simple ways in which breeders can use our model to assess the value of enhanced leaf cooling as a criterion for selection in a warming climate. Using growing season weather data, breeders can use our model to assess differences in the duration of threshold exceedance between Calima and SAB 686 in within sample TPEs. Estimates of threshold exceedence could be focused on micro- and macro-sporogenesis, when the plant is particularly sensitive to high temperatures. Breeders can also use our model to estimate the accumulated impact of differences in leaf cooling over the course of the growing season in within sample TPEs. For example, breeders could use our model in conjunction with growing season weather data to estimate genotypic differences in growing degree days from differences in leaf cooling. Breeders could build similar models for a variety of TPEs to explore the potential benefits of enhanced leaf cooling across bean growing regions.

Breeders could use the methods we have demonstrated in this paper to build low input G × E models of leaf temperature in crop growth models. Doing so would allow breeders to assess the emergent impacts of G × E interactions in leaf cooling on complex traits like yield at the system level (Bertin et al., 2010). Theoretically, crop growth models could also be used to study the trade-offs between greater leaf cooling in different TPEs. Integrating genotype specific equations for leaf temperature in crop growth models could help breeders to quantify trade-offs between selecting for enhanced leaf cooling in hot irrigated environments and depletion of available soil water in hot, dry and rainfed environments.

In addition to helping breeders to understand the system-wide implications of genotypic differences in leaf cooling, our results support the argument that simulating the temperature of the leaf/canopy would improve heat stress assessments. An argument that has also been made for other crops (Webber et al., 2016) as well as for land-surface vegetation modeling (Dong et al., 2017). However, the scale of this task should not be underestimated. Crop growth models often use air temperature in growth and phenology functions (Neukam et al., 2016), and these would need to be re-written using leaf/canopy temperatures. Furthermore, in a comprehensive multi-model study testing crop model skill at simulating canopy temperature, Webber et al. (2018) show that the best performing models were able to explain only 30%–40% of variance in the difference between leaf and air temperatures (Webber et al., 2018).

The success of this endeavor will depend on the availability of sufficient data and further testing of empirical methods across the wide range of environments in which crop models need to perform. The recent uptake of MultispeQ devices with an open source data platform suggests that data availability will be forthcoming. However, our findings of a within canopy gradient suggest that future experiments aimed at understanding the impacts of leaf cooling should also consider how temperatures vary within the canopy.

### Limitations

We have explored phenotypic differences in leaf cooling during the daytime across three contrasting genotypes. However, beans are also sensitive to high nighttime temperatures. Further research needs to test if heat tolerant genotypes also cool more at night when stomatal conductance is close to zero and overall transpiration is more limited. This will allow us to decouple the impacts of transpirational cooling from differences in leaf traits and to explore potential differences in the cost of nighttime respiration.

We have shown that leaf temperature can be accurately and usefully modeled using only temperature and relative humidity in irrigated conditions. However, this does not necessarily imply that the same will be true under varying water-limited scenarios. We are currently conducting experiments measuring the same set of environmental variables under varying conditions of water availability. In future work, we will use the results of these ongoing experiments to see if leaf temperature under water limiting conditions can also be simply modeled with high accuracy. A further limitation of our modeling approach is that we do not include solar radiation as a variable. We made this choice because solar radiation is often highly correlated with air temperature, so it is not advisable to use both variables in the same model. We chose to use temperature because it is more widely available from weather stations in the TPEs in which we conduct breeding work. Our results suggests that this formulation works well in environments with very large variation in solar radiation (field vs. greenhouse). However, there are likely to be interaction effects between temperature, relative humidity and solar radiation and new versions of the model may be needed for TPEs with contrasting solar radiation, VPD and soil water availability. This will be explored in future work.

To fully ascertain the importance of variation in leaf cooling between heat tolerant and heat sensitive genotypes, we will need to explore the impacts of greater leaf cooling on yield quality and quantity under multiple target population of environments. Tardieu (2011) highlights that traits which confer tolerance in one set of environmental conditions can confer sensitivity under different conditions. For example, genotypes which increase heat avoidance through enhanced cooling may confer tolerance under hot and irrigated conditions. This same trait could induce sensitivity under hot and dry conditions, through early depletion of available soil water. Future work will need to use models to understand the trade-offs inherent in enhanced leaf cooling in changing target population of environments of the future. This will allow the costs and benefits of breeding for enhanced transpirational cooling to be more realistically assessed.

### Summary

We have shown that leaf cooling plays an important role in heat avoidance. Heat tolerant genotypes cool their leaves more than heat sensitive genotypes, and this difference increases under hot and dry conditions. Furthermore, the difference in leaf cooling is largest at the top of canopy where determinate bush beans are most sensitive to high temperatures during the flowering period. We have shown that heat tolerant varieties exhibited higher stomatal conductance and a greater association between VPD and leaf cooling. This suggests that the heat tolerant genotype cooled more because of enhanced transpirational cooling. Leaf thickness may also have played a role, but differences in thickness were not large enough to prove this conclusively.

Our work suggests that bean breeders can use LTD to screen for beans with enhanced capacity for heat avoidance. Future work will need to test this conclusion with more genotypes and in a wider range of environmental conditions. We have shown that it is possible to simulate leaf temperature by genotype accurately. Future work will need to explore the success of the empirical methods used in this paper with a wide range of genotypes across target population of environments. In particular, it will be important to explore model performance under conditions with contrasting VPD, solar radiation and soil water availability. Our results suggest that expanding this modeling approach to assess the value of enhanced transpirational cooling across target population of environments has the potential to directly inform bean breeding programs.

## Data Availability Statement

The datasets generated for this study can be found in the PhotosynQ repository: https://photosynq.org/projects/ciat-bean-high-nighttime-temperatures-18-01-4089, https://photosynq.org/projects/ciat-bean-high-day-and-nighttimetemperature-18-12-5578, https://photosynq.org/projects/ciat-bean-soil-vs-cylinders-under-heat-stress-18-18-3767, https://photosynq.org/projects/ciat-bean-soil-vs-cylinders-and-heat-stress-18-18-3768, https://photosynq.org/projects/ciat-bean-leaf-prolongation-under-drought-18-08-4747, https://photosynq.org/projects/ciat-bean-rice-bean-rotation-soil-compaction-18-03-4169. A free account can be made at the website in order to view the datasets.

## Author Contributions

CD conceived the study, performed the analysis, and wrote the paper. MU performed the experiments and provided guidance on using the observations. MU also provided feedback as the paper took form and commented on draft versions of the paper. AC provided regular feedback as the paper took form and provided discussion and feedback on draft versions of the paper. PF provided regular feedback as the paper took form and provided discussion and feedback on draft versions of the paper. LS conducted the specific leaf area measurements for experiment D2, explained the protocols and instrumentation used and provided guidance on using and interpreting these measurements. LS also provided discussion and feedback on draft versions of the paper.

## Funding

CD was supported by a UK National Environmental Research Council Industrial Case Award in collaboration with the UK Met Office NE/M009793/1. AC is supported by the CGIAR Research Program on Climate Change, Agriculture and Food Security (CCAFS), which is carried out with support from CGIAR Fund Donors and through bilateral funding agreements. For details please visit https://ccafs.cgiar.org/donors. PF was supported by the Met Office Hadley Centre Climate Programme funded by BEIS and Defra. MU was supported by a Biotechnology and Biological Sciences Research Council (BBSRC) funded project named Bean Breeding for Adaptation to a Changing Climate and Post-Conflict Colombia (BBACO). Grant number BB/S018964/1. The views expressed in this document cannot be taken to reflect the official opinions of these organizations.

## Conflict of Interest

The authors declare that the research was conducted in the absence of any commercial or financial relationships that could be construed as a potential conflict of interest.
