# Supplementary material for: Enhanced Leaf Cooling Is a Pathway to Heat Tolerance in Common Bean
Source: Front Plant Sci. 2020 Feb 28;11:19. doi: 10.3389/fpls.2020.00019 (PMC7059850; doi:10.3389/fpls.2020.00019)
Supplement: Supplementary file 1 [file DataSheet_1.pdf]

Supplementary Material

Table 1: Summary of Experiments

Experiments	Treatments	No. observations in whole sample analysis	Days Sampled	Genotypes	Location	Irrigation	Season
H1	Ambient, GH control, GH night heat	3306	10	3	Cali, Colombia	Irrigated	Spring 2018
H2	Ambient, GH night heat	832	3	10	Cali, Colombia	Irrigated	Summer 2017
H3	Ambient, GH control, GH night heat	266	3	3	Cali, Colombia	Irrigated	Spring 2018
D1	Ambient, Early Drought, Late Drought	1287	1	20	Cali, Colombia	Full, Partial, Partial	Summer 2018
S1	Soil compaction from rice rotation	1066	2	6	Cali, Colombia	Irrigated	Spring 2018

Experiment	Test	Treatment	Test-statistic	p-value
H1	Calima vs. SAB 686	Ambient	two-sided permutation test	0.980
H1	Calima vs. SEF 60	Ambient	two-sided permutation test	0.576
H1	Calima vs. SAB 686	Greenhouse control	two-sided permutation test	0.608
H1	Calima vs. SEF 60	Greenhouse control	two-sided permutation test	0.431
H1	Calima vs. SAB 686	Greenhouse night heat	two-sided permutation test	0.205
H1	Calima vs. SEF 60	Greenhouse night heat	two-sided permutation test	0.062
H2	Calima vs. SAB 686	Ambient	two-sided permutation test	0.836
H2	Calima vs. SAB 686	Greenhouse night heat	two-sided permutation test	0.398

Table 2: Test statistics for a comparison of leaf angle group means between genotypes for each treatment of the H1 and H2 experiments.

Experiment	Test	Treatment	Test-statistic	p-value
H1	Calima vs. SAB 686	Ambient	two-sided permutation test	0.012
H1	Calima vs. SEF 60	Ambient	two-sided permutation test	0.045
H1	Calima vs. SAB 686	Greenhouse control	two-sided permutation test	0.113
H1	Calima vs. SEF 60	Greenhouse control	two-sided permutation test	0.053
H1	Calima vs. SAB 686	Greenhouse night heat	two-sided permutation test	0.081
H1	Calima vs. SEF 60	Greenhouse night heat	two-sided permutation test	0.900
H2	Calima vs. SAB 686	Ambient	two-sided permutation test	0.886
H2	Calima vs. SAB 686	Greenhouse night heat	two-sided permutation test	0.358

Table 3: Test statistics for a comparison of leaf thickness group means between genotypes for each treatment of the H1 and H2 experiments.

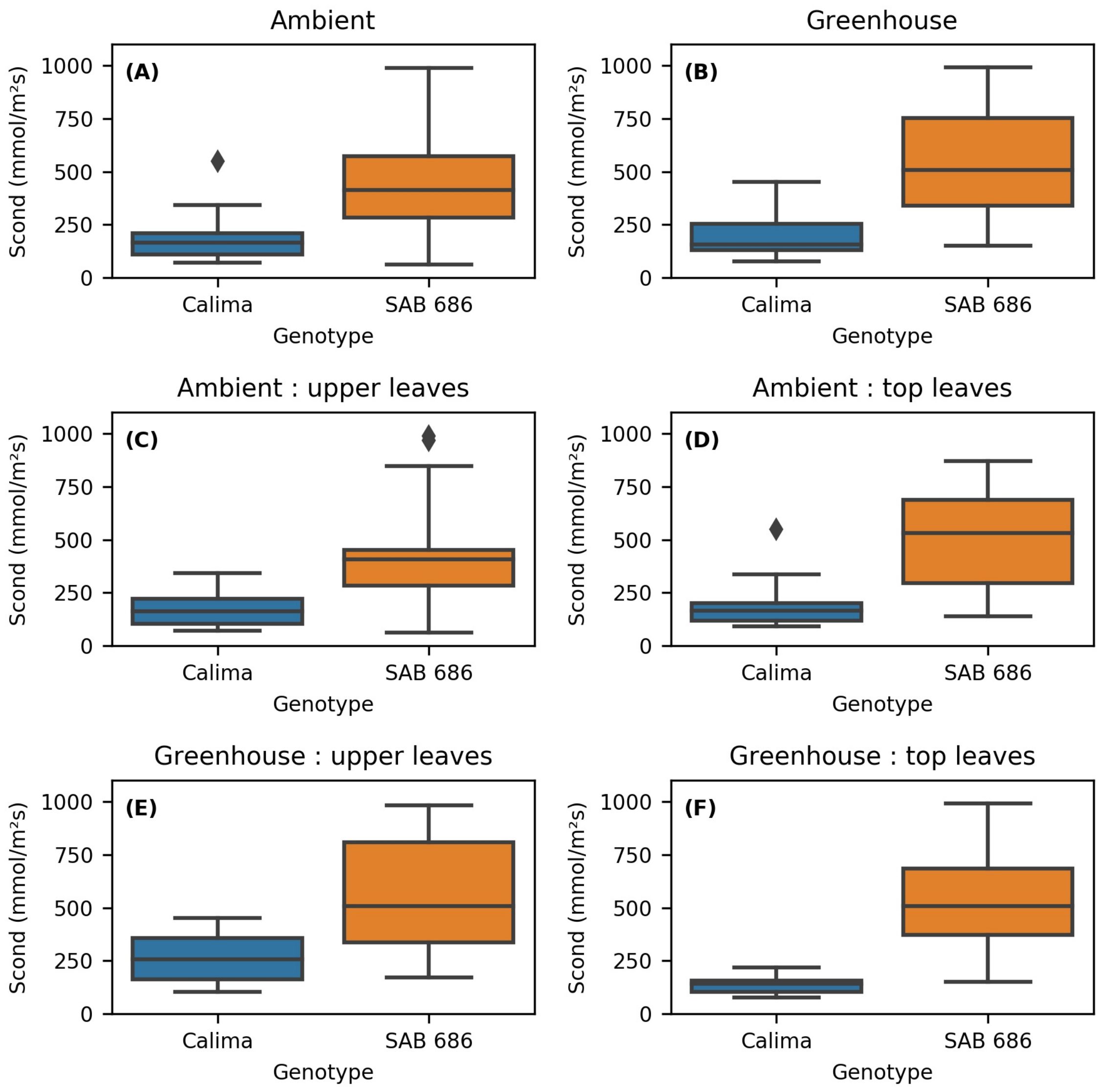

Figure 1: The distribution of stomatal conductance for Calima and SAB686 for experiment H2 (A) Ambient treatment (B) Greenhouse night heat treatment (C) Ambient treatment-upper leaves (D) Ambient treatment-top leaves (E) Greenhouse night heat treatment-upper leaves (F) Greenhouse night heat treatment-top leaves

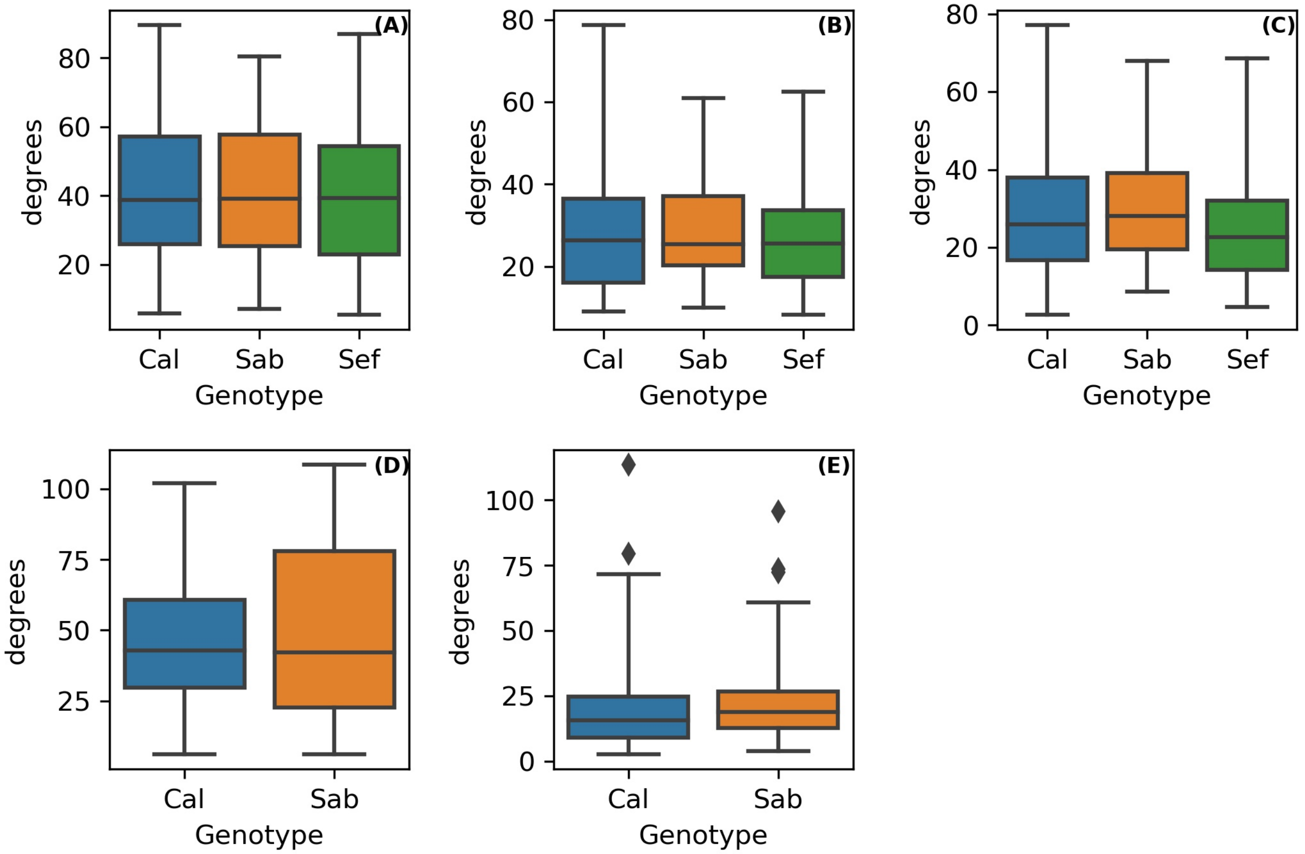

Figure 2: The distribution of leaf angle by genotype in experiments H1 and H2 (A) H1-ambient treatment (B) H1-greenhouse control treatment (C) H1-greenhouse night heat treatment (D) H2-ambient treatment (E) H2-greenhouse night heat treatment

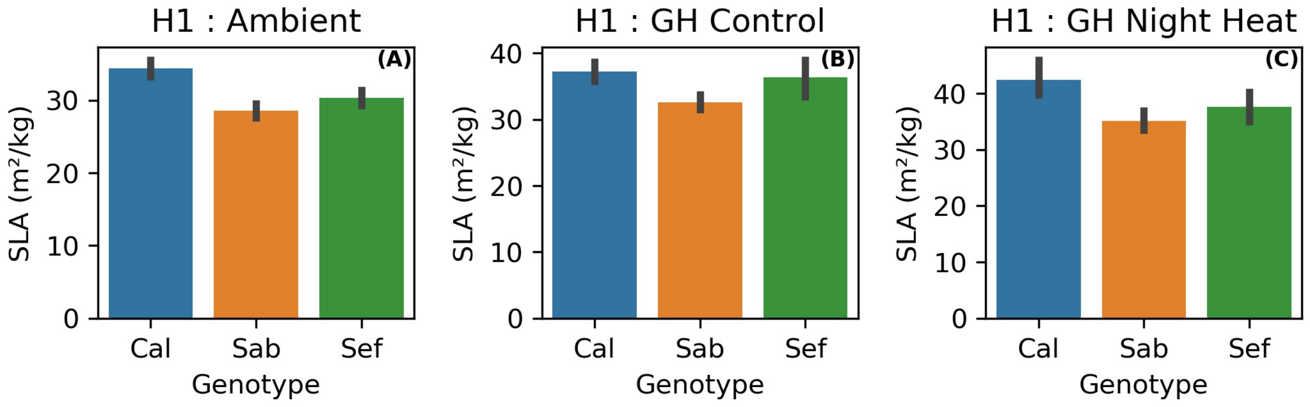

Figure 3: Mean Specific Leaf Area by genotype in experiment H1 (A) Ambient treatment (B) Greenhouse control treatment (C) Greenhouse night heat treatment. Error bars represent the 95th confidence interval

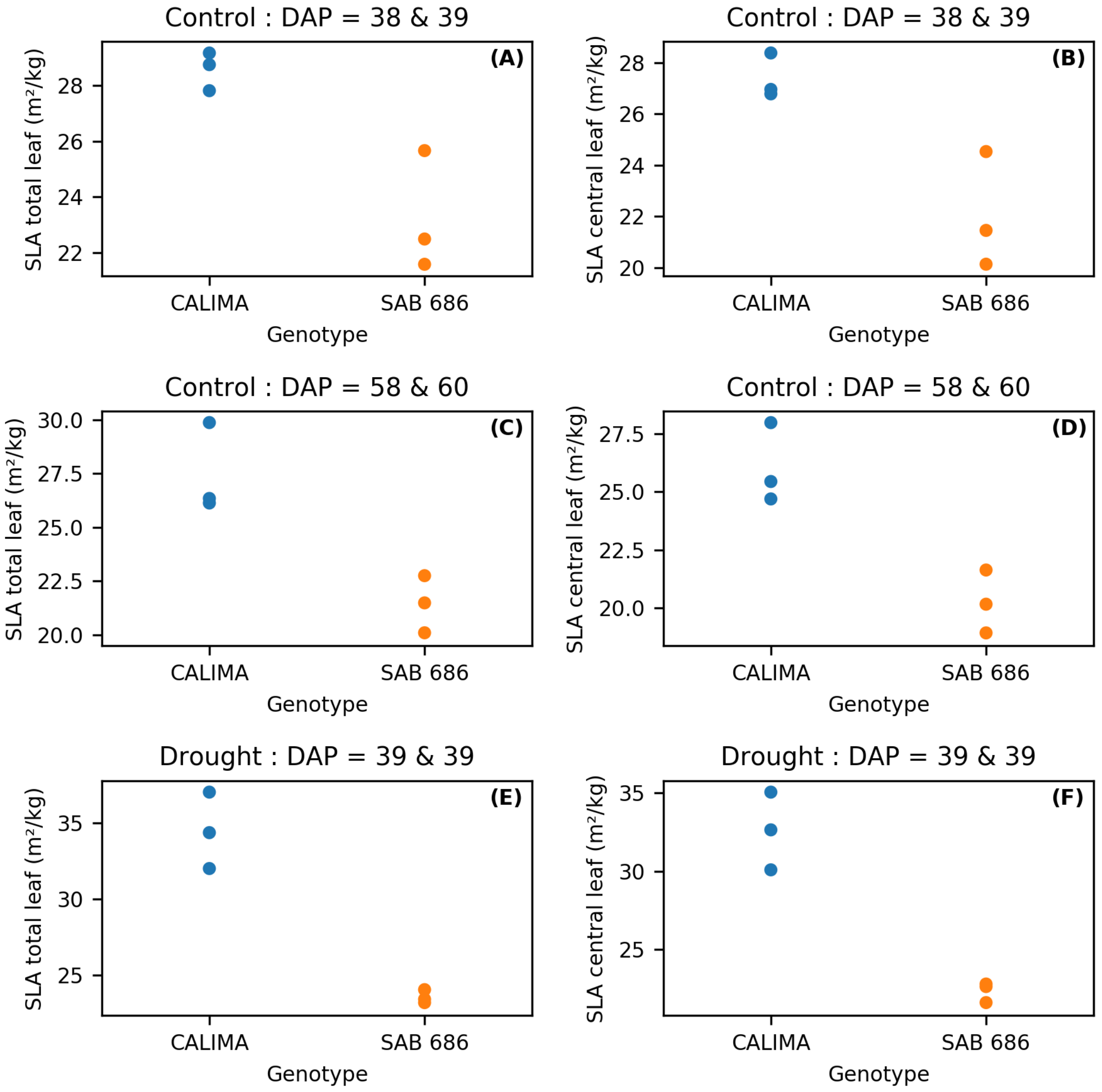

Figure 4: Specific Leaf Area by genotype in experiment D2 for the whole trifoliate leaf and the central trifoliate leaf (A), (C) and (E) gives the SLA of the whole trifoliate leaf during snapshots of the control and drought treatments. (B), (D) and (F) gives the SLA of the central trifoliate leaf during snapshots of the control and drought treatments. DAP is short for days after planting. Each dot represents the average of 5 replications

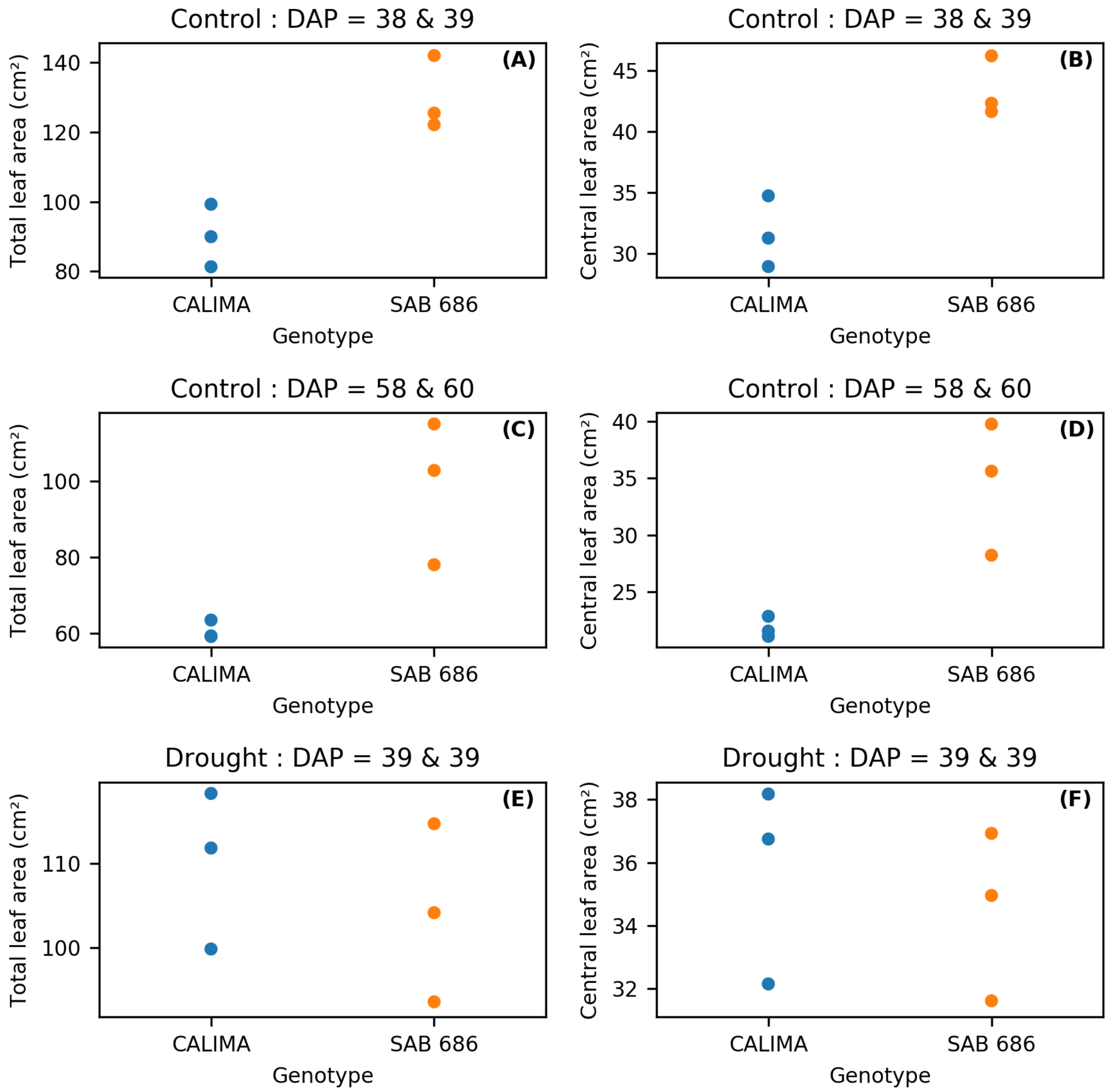

Figure 5: Leaf Area by genotype in experiment D2 for the whole trifoliate leaf and the central trifoliate leaf (A), (C) and (E) gives the LA of the whole trifoliate leaf during snapshots of the control and drought treatments. (B), (D) and (F) gives the LA of the central trifoliate leaf during snapshots of the control and drought treatments. DAP is short for days after planting. Each dot represents the average of 5 replications

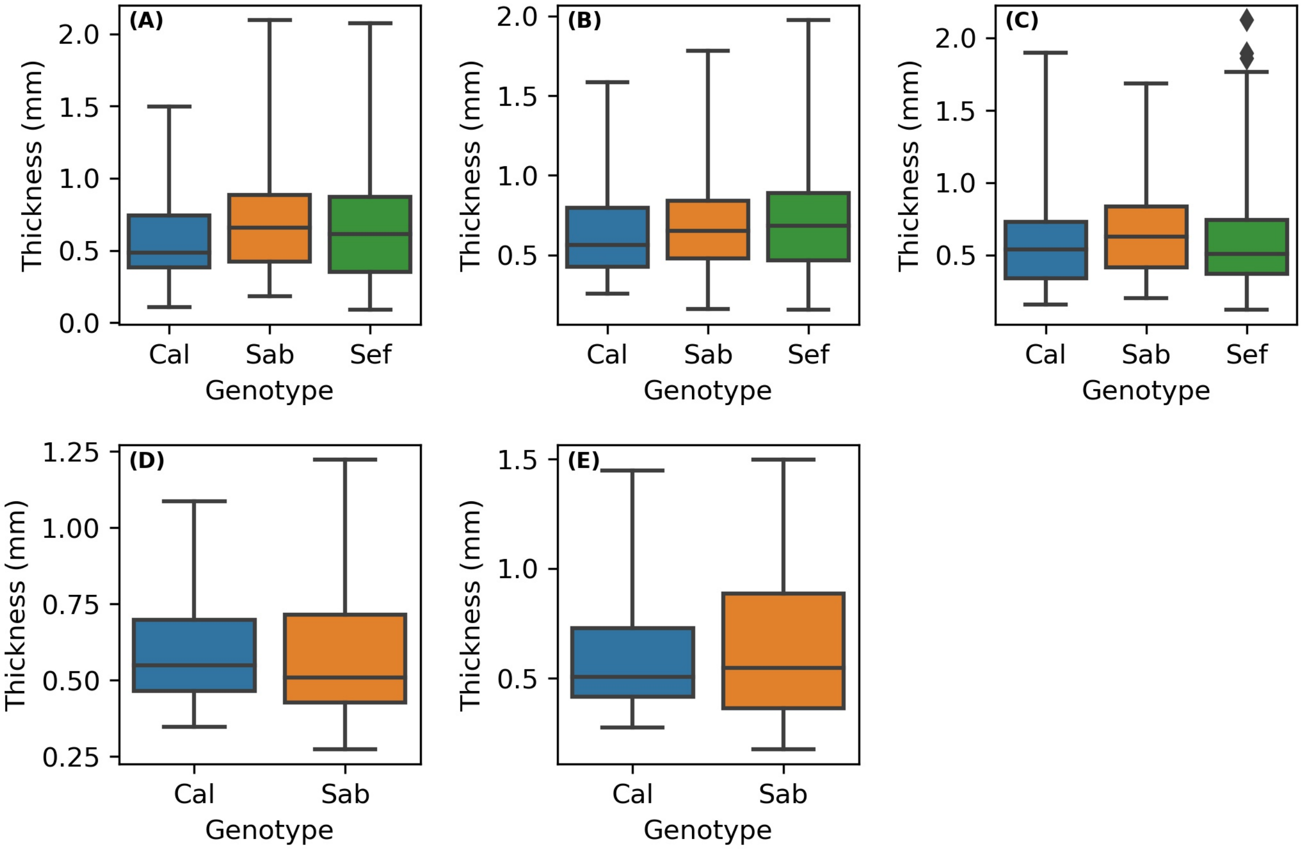

Figure 6: Distribution of leaf thickness by genotype in experiments H1 and H2 (A) H1-ambient treatment (B) H1-greenhouse control treatment (C) H1-greenhouse night heat treatment (D) H2-ambient treatment (E) H2-greenhouse night heat treatment
